# The structure of human dermatan sulfate epimerase 1 emphasizes the importance of C5-epimerization of glucuronic acid in higher organisms†
†Electronic supplementary information (ESI) available. See DOI: 10.1039/d0sc05971d


**DOI:** 10.1039/d0sc05971d

**Published:** 2020-12-08

**Authors:** Mahmudul Hasan, Hamed Khakzad, Lotta Happonen, Anders Sundin, Johan Unge, Uwe Mueller, Johan Malmström, Gunilla Westergren-Thorsson, Lars Malmström, Ulf Ellervik, Anders Malmström, Emil Tykesson

**Affiliations:** a Department of Biochemistry and Structural Biology , Lund University , Lund , Sweden; b Equipe Signalisation Calcique et Infections Microbiennes , Ecole Normale Supérieure Paris-Saclay , 91190 Gif-sur-Yvette , France; c Institut National de la Santé et de la Recherche Médicale U1282 , 91190 Gif-sur-Yvette , France; d Department of Clinical Sciences , Lund University , Lund , Sweden; e Department of Chemistry , Lund University , Lund , Sweden; f Department of Experimental Medical Science , Lund University , Lund , Sweden . Email: emil.tykesson@med.lu.se; g Macromolecular Crystallography Group , Helmholtz-Zentrum-Berlin für Materialien und Energie , Albert-Einstein Str. 15 , 12489 Berlin , Germany; h Department of Biological Chemistry , University of California Los Angeles , Los Angeles , CA 90095 , USA

## Abstract

Dermatan sulfate epimerase 1 (DS-epi1, EC 5.1.3.19) catalyzes the conversion of d-glucuronic acid to l-iduronic acid on the polymer level, a key step in the biosynthesis of the glycosaminoglycan dermatan sulfate. Here, we present the first crystal structure of the catalytic domains of DS-epi1, solved at 2.4 Å resolution, as well as a model of the full-length luminal protein obtained by a combination of macromolecular crystallography and targeted cross-linking mass spectrometry. Based on docking studies and molecular dynamics simulations of the protein structure and a chondroitin substrate, we suggest a novel mechanism of DS-epi1, involving a His/double-Tyr motif. Our work uncovers detailed information about the domain architecture, active site, metal-coordinating center and pattern of *N*-glycosylation of the protein. Additionally, the structure of DS-epi1 reveals a high structural similarity to proteins from several families of bacterial polysaccharide lyases. DS-epi1 is of great importance in a range of diseases, and the structure provides a necessary starting point for design of active site inhibitors.

## Abstract 2

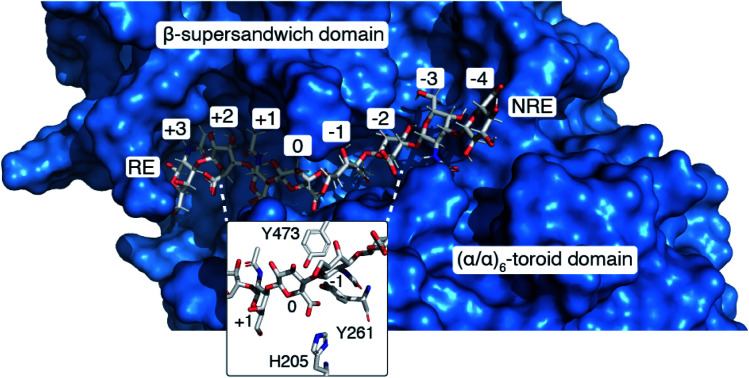
Structural studies of human DS-epi1 suggests a new catalytic isomerization mechanism and reveals remarkable similarities to bacterial proteins.

## Introduction

**Fig. 1:**
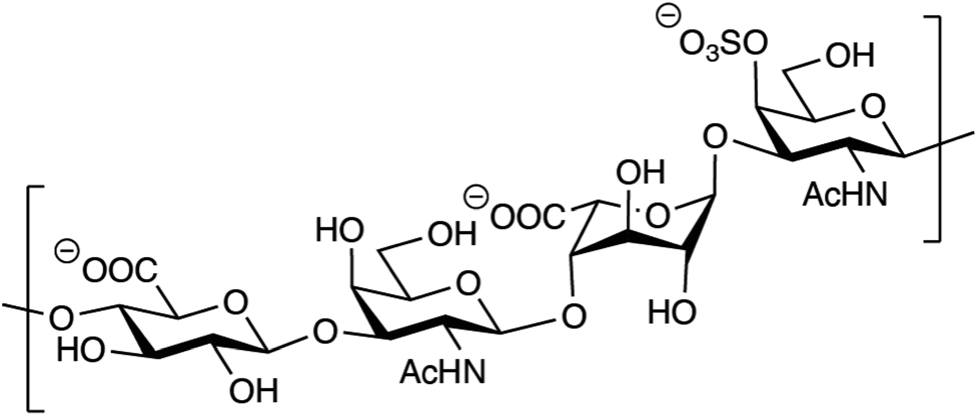
Structure of a theoretical CS/DS tetrasaccharide with the sequence GlcAβ1–3GalNAcβ1–4IdoAα1–3GalNAc,4S.

Out of the four major classes of biological macromolecules: nucleic acids, proteins, lipids and carbohydrates, the latter is arguably the most complex and least understood. Notably, the group of linear, heterogeneous polysaccharides called glycosaminoglycans (GAGs) are synthesized in a non-template-driven manner, but still carry distinct sequences crucial for biological interactions.^1^–^3^ The GAGs are most often found as part of proteoglycans, consisting of one or more GAG chains covalently attached to a protein core. One of the most common GAG types, chondroitin/dermatan sulfate (CS/DS), is built up by repeating disaccharide units of d-glucuronic acid (GlcA) and *N*-acetyl-d-galactosamine (GalNAc). The CS/DS polymer can be modified by epimerization and sulfation, introducing the potential for vast structural diversity (Fig. 1).^4^ The two human dermatan sulfate epimerases DS-epi1 and DS-epi2, essential for the epimerization of position 5 of GlcA residues to form l-iduronic acid (IdoA), were previously identified, cloned, expressed, purified and functionally evaluated by our group.^5^,^6^

We, and others, have previously shown that the patterns of epimerization and sulfation of CS/DS is of critical importance for a range of biological activities.^7^ For example, patients diagnosed with Ehlers–Danlos syndrome caused by loss-of-function mutations in DS-epi1 suffer from multiple organ disorders.^8^–^11^ Furthermore, IdoA-containing motifs are important in processes such as control of collagen fibril formation, cancer development, thrombin inactivation through heparin cofactor II (HCII), growth factor interactions as well as for cell migration.^12^–^17^

The formation of IdoA in CS/DS, by epimerization of GlcA on the polymer level, is an interesting exception to the biosynthesis of most other polysaccharides, which are synthesized from a pool of activated monosaccharide donors. In fact, to date there are only three known examples of post polymerization epimerization, *i.e.* the biosyntheses of alginate, heparin/heparan sulfate and CS/DS.^18^ Despite the resemblances on the functional level, the amino acid sequences of the epimerases show no similarity on the primary level. By performing domain prediction and profile–profile alignments with sequence profiles of proteins from PDB, COG, Pfam and SCOP, we have previously shown that DS-epi1 has no domain similarity to the alginate and heparan sulfate epimerases but is remotely related to polysaccharide-degrading bacterial enzymes.^19^ These findings suggested the location of the active site and catalytic amino acids of DS-epi1 and were further supported by site-directed mutagenesis studies. However, so far, no experimental structural data has been presented for DS-epi1 or DS-epi2.

In this study we present the three-dimensional structure of human *N*-glycosylated DS-epi1, solved by a combination of macromolecular crystallography and targeted cross-linking mass spectrometry (TX-MS).^20^ The structure reveals a high similarity to proteins from several families of bacterial polysaccharide lyases. By using a combination of *in silico* docking and molecular dynamics simulations, we also propose a novel catalytic mechanism for DS-epi1.

## Results

### Human DS-epi1 is built up by an (α/α)_6_-toroid domain connected to a β-supersandwich domain

**Fig. 2:**
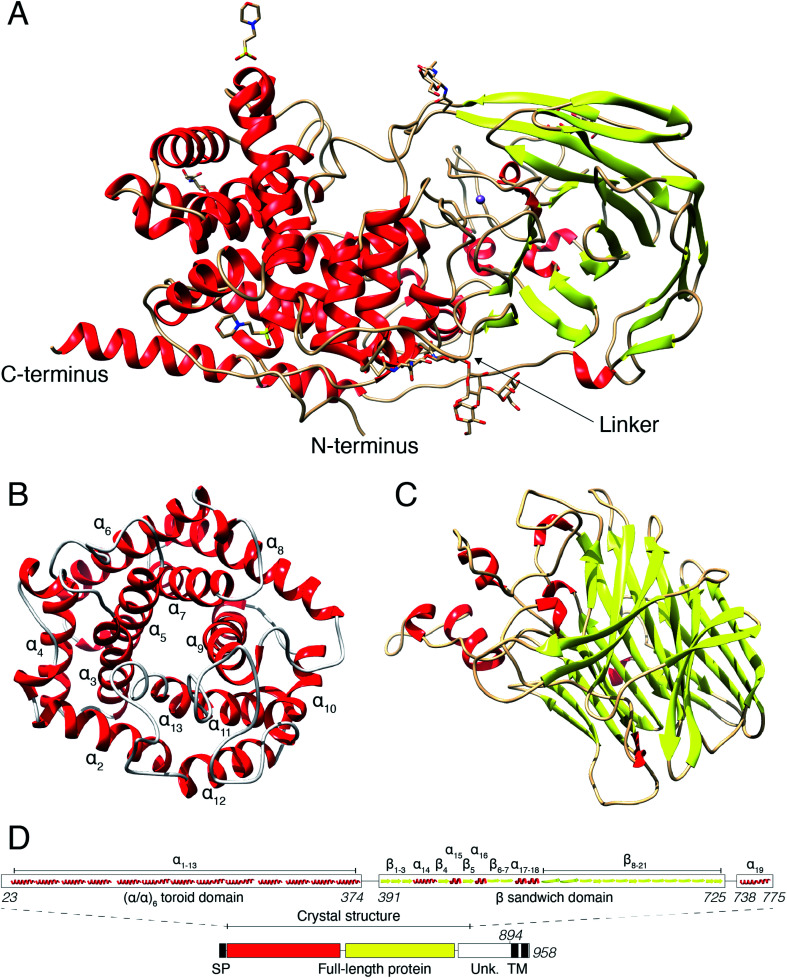
Crystal structure of human DS-epi1. (A) Overview of the DS-epi1 crystal structure consisting of 743 amino acids, nine monosaccharides, two MES buffer molecules and one manganese atom. The N- and C-termini are highlighted, as well as the linker connecting the two main domains. (B) Isolated view of the cone shaped (α/α)_6_-toroid domain. (C) Isolated view of the β-supersandwich domain. (D) Schematic representation of the full-length protein (bottom) and the crystallized part (top). SP – signal peptide, TM – trans-membrane domains, Unk – domain of unknown structure.

In a previous study, we revealed that the first 755 amino acids of DS-epi1 are necessary for catalytic activity.^21^ Based on those results, we used five constructs of ≥755 amino acids, spanning the C-terminal domain of unknown function, for protein expression attempts in mammalian HEK293 GnTI(–) cells.^22^ Only one construct, corresponding to amino acids 23–775 (DS-epi1 23–775), yielded diffraction-quality crystals, but still displayed a catalytic profile similar to that of the full-length luminal protein (DS-epi1 23–894), with only slight variations in *K*_M_ and *V*_max_ (ESI Fig. 1A†). The truncated soluble DS-epi1 23–775 protein had a molecular weight of 89.2 kDa and was pure, monomeric and monodispersed (ESI Fig. 1B and C†). We have previously shown that DS-epi1 forms homodimers *in vivo*,^23^ and therefore wanted to evaluate if dimerization occurs in a concentration-dependent manner. Both full-length luminal DS-epi1 23–894 and truncated DS-epi1 23–775 were analyzed using size-exclusion chromatography at different concentrations between 0.1–12 mg ml^–1^ (ESI Fig. 1D and E†), but no sign of dimerization could be seen at any concentration. Crystals of DS-epi1 23–775 grew after approximately one week of incubation at 22 °C (ESI Fig. 1F†). The single-wavelength anomalous dispersion (SAD) method, using the anomalous signal from manganese, produced a partial model of DS-epi1 (see Table 1 for data collection and refinement statistics). Using the derived model, a more complete crystallographic structure was built using a 2.41 Å dataset and thereafter refined to an *R* factor of 17.6% (*R*_free_ 20.9%). Each unit cell contained eight protein molecules and had a relatively high solvent content of 72%. A majority of the structure could be built, except nine C-terminal residues which were not visible in the electron density, revealing two large domains connected by an 18 amino acid linker loop, and a C-terminal tail extending back to the N-terminus (Fig. 2A). The linker loop contains three adjacent prolines (Pro382–384), likely contributing to flexibility of the two domains. Accordingly, mutagenesis of Pro383 to alanine yielded an enzymatically inactive product which was expressed at very low levels (ESI Fig. 2†). The N-terminal domain was clearly defined in the electron density, however, with *B* factor values around 50. In contrast, the C-terminally located domain was overall more disordered, and specific loop regions, especially amino acids 507–515, were poorly detailed in the densities with substantially higher *B*-factors. The N-terminal domain (Fig. 2B) was identified as an (α/α)_6_-toroid domain (amino acids 65–373, SCOP superfamily a.102.3), while the C-terminally located domain (Fig. 2C) was identified as a 4-sheet β-supersandwich domain (amino acids 391–725, SCOP superfamily b.30.5). Most of the unknown C-terminal domain (amino acids ∼740–894) was truncated in the crystallization construct and is only visible in the structure as a 45 Å long α helix, connected to the central domain *via* a loop consisting of 12 residues. Based on the structure, a HERA^24^ topology scheme was generated (ESI Fig. 2†) using PDBsum,^25^ and each secondary element was labelled with unique identifiers (Fig. 2D). On the gene level, DS-epi1 is encoded by the DSE gene, consisting of six exons with the coding sequence extending from exon 2–6. As revealed by the crystal structure, helices α1–α4 are encoded by exon 2, α5–α7 by exon 3, α8–α10 by exon 4, α11–α13 by exon 5 (*i.e.* the N-terminal alpha-toroid domain is encoded by exon 2–5) whereas the final part of the polypeptide, including the β-sandwich domain and the C-terminal non-crystallized part, is encoded by exon 6.

**Table 1:** Data collection and refinement statistics. Statistics for the highest-resolution shell are shown in parentheses

	Native data	Anomalous data
Wavelength	0.97967	1.89288
Resolution range (Å)	48.57–2.41 (2.496–2.41)	48.60–3.60 (3.729–3.6)
Space group	*C*222_1_	*C*222_1_
Unit cell (Å, °)	182.676, 213.914, 86.945, 90, 90, 90	184.098, 210.596, 85.975, 90, 90, 90
Total reflections	694 344 (47 028)	191 807 (46 241)
Unique reflections	66 029 (4393)	19 795 (1448)
Multiplicity	10.5 (10.7)	9.7 (10.0)
Anomalous multiplicity	—	5.0 (5.0)
Completeness (%)	100.0 (100.0)	99.9 (100.0)
Anomalous completeness (%)	—	99.9 (99.9)
Mean I/sigma (I)	15.71 (1.03)	10.1 (3.1)
Wilson *B*-factor (Å^2^)	67.29	114.05
*R*_merge_ (I)	0.092 (2.729)	0.160 (0.712)
*CC*_1/2_	0.999 (0.407)	0.997 (0.934)
*R*-Work	0.1756 (0.3129)	
*R*-Free	0.2089 (0.3470)	
Number of non-hydrogen atoms	6304	
Macromolecules	6009	
Ligands	142	
Solvent	153	
Protein residues	743	
RMSD (bonds, Å)	0.005	
RMSD (angles, °)	1.13	
Ramachandran favored (%)	95.68	
Ramachandran outliers (%)	0.67	
Clashscore	4.40 (100^th^ percentile)	
Average *B*-factor (Å^2^)	79.90	
Macromolecules	79.28	
Ligands	114.36	
Solvent	72.12	

### A highly conserved core of DS-epi1 harbors a manganese-binding site and the suggested active site

**Fig. 3:**
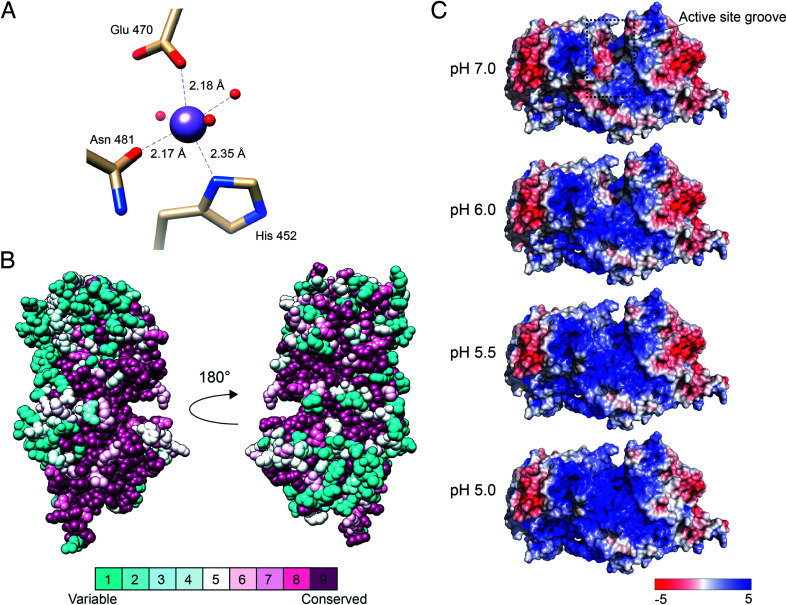
Manganese-binding site, conservation among eukaryotic orthologs and electrostatic surface potential of DS-epi1. (A) Manganese-binding site of DS-epi1. The manganese ion and water oxygens are shown as spheres while interacting residues are depicted as sticks and colored according to different atom types (beige, carbon; red, oxygen; blue, nitrogen). Amino acid–ion bonds are shown as dashed lines with bond lengths specified adjacently. (B) The amino acid conservation of human DS-epi1 among eukaryotic orthologs (ESI Table 1†) was analyzed using the ConSurf server. The results were visualized using spheres colored going from least (turquoise) to most (maroon) conserved on a relative scale. (C) The surface electrostatic potential of DS-epi1 contoured at ±5 *kT*/*e*. A positive potential appears in the suggested substrate binding groove between pH 5–6, correlating with the pH optimum of DS-epi1. Calculations were made using the APBS electrostatics plugin of PyMOL version 2.3.2. PQR input files calculated for different pH were prepared using the PDB2PQR server (; http://nbcr-222.ucsd.edu/pdb2pqr_2.1.1/).

Situated in the central beta sandwich domain, a strong peak was observed in the initial SAD electron density map. Based on the previous knowledge of the necessity of Mn^2+^ for catalytic activity,^26^ 2 mM MnCl_2_ was included in the crystallization reservoir. Tuning the wavelength during data collection to the absorption edge of manganese resulted in a stronger signal and the density was identified as a divalent manganese ion. The manganese was found to coordinate octahedrally with nitrogen and oxygen atoms of three amino acids (His452, Glu470 and Asn481) and three water molecules (H_2_O 25, H_2_O 27 and H_2_O 34) (Fig. 3A). The coordination distances between the Mn^2+^ ion and the liganding atoms were measured to be: ND1^His452^, 2.35 Å; OE1^Glu470^, 2.18 Å; OD1^Asn481^, 2.17 Å; H_2_O25, 2.19; H_2_O^27^, 2.20; H_2_O^34^ 2.19 Å, similar to the regular range of metal coordination distances observed in protein structures.^27^ Mutagenesis of the Mn-coordinating amino acids either completely abolished the catalytic activity or did not yield an expressed protein (ESI Fig. 3†).

The suggested active site of DS-epi1 is found buried deeply between the alpha-toroid and the beta-sandwich domains. Using a list of eukaryotic orthologs of DS-epi1 (ESI Table 1†), we analyzed the amino acid conservation of DS-epi1 using the ConSurf server.^28^ The results (Fig. 3B) revealed that the active site cleft, as well as parts of the C-terminal tail, were highly conserved, further supporting the location of the active site.

DS-epi1 is active over a pH range from 5 to 7, with an optimum of 5.5.^26^ Using the crystal structure as input, we investigated how different pH values affected the electrostatic surface potential of DS-epi1. The active site was found to harbor an overall negative electrostatic potential at pH 7.0, which gradually changed into a positive charge as the pH was decreased (Fig. 3C).

### Docking studies with a chondroitin octasaccharide

**Fig. 4:**
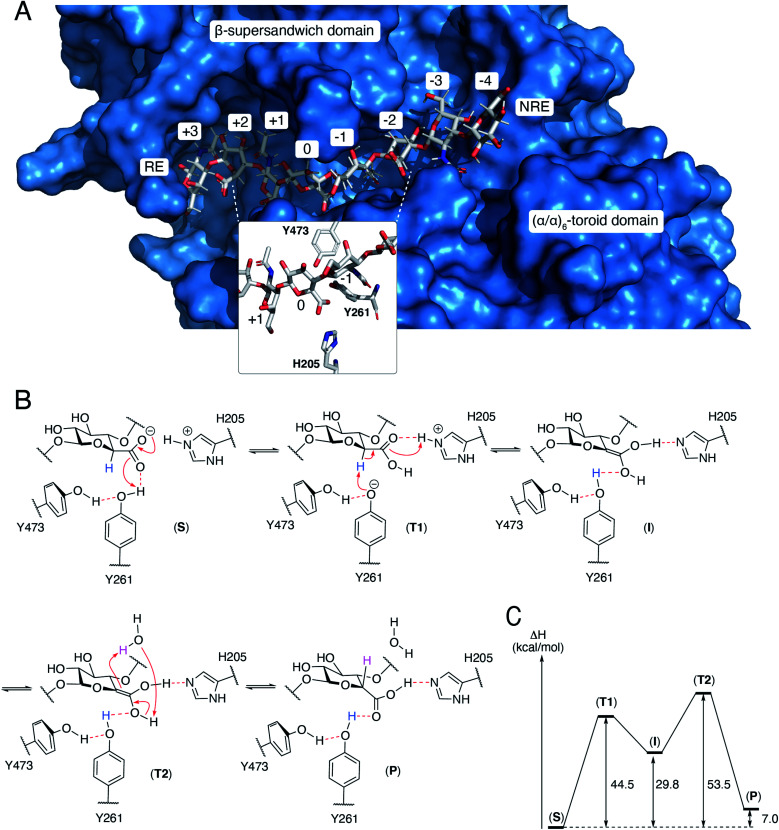
(A) A docked octasaccharide in the curved cleft of DS-epimerase 1, showing numbering of carbohydrate residues of the octasaccharide and the amino acids of the active site. RE = reducing end, NRE = non-reducing end. (B) Proposed mechanism of DS-epi1. The C5 proton abstracted from GlcA is colored in blue and the proton transferred to C5 of IdoA is colored in magenta. All electron movements are shown with red arrows. (C) Energies from quantum mechanics calculations. S = substrate, T = transition state, I = intermediate, P = product.

The N-terminal domain and the central domain of DS-epimerase 1 form a deep, curved cleft with a length of approximately 40 Å, that constitutes the carbohydrate binding domain. Despite extensive co-crystallization and soaking attempts, using different crystallization conditions, substrate analogues and point mutants, no co-crystal was obtained. Hence, to study the binding and interactions of DS-epi1 with a substrate oligosaccharide, we docked a chondroitin octasaccharide in the presumed active site. In addition to the regular six degrees of freedom of a rigid molecule, an octameric oligosaccharide has 14 rotatable glycosidic torsions, making it a complicated problem for any docking program. Therefore, chondroitin di- and tetrasaccharides were placed at different positions along the carbohydrate binding domain followed by molecular dynamics simulations. A tetrasaccharide found a stable binding pose adjacent to the catalytic site and this tetrasaccharide was extended into and past the catalytic site, one carbohydrate at a time, followed by new molecular dynamics simulations. The carbohydrate units were numbered, with reference from the GlcA unit situated at the catalytic site, from the reducing end (+3) to the nonreducing end (–4) (Fig. 4A).

The resulting ligand conformation featured the GalNAc adjacent to the reacting GlcA, *i.e.* position –1, with a hydrophobic interaction with Trp98, similar to that of galactose recognition in galectins^29^ (see ESI Video 1 and Coordinate File 1†). In line with the docking results, a Trp98Ala mutant had a reduced activity of almost 90% (ESI Fig. 3†). The carboxyl groups of all GlcA were pointing towards the water phase, except for the one undergoing isomerisation, which was hydrogen bonded by protonated His205 and by Tyr261, supported by a hydrogen bond from Tyr473. In close proximity to the active site, His450, previously thought to act as a general base for abstraction of the C5 hydrogen of the uronic acid, formed hydrogen bonds with the carboxylate group of GlcA *via* a water bridge.

### Theozyme model of the isomerization mechanism

A theozyme is a QM theoretical model of an enzyme, only including the parts of the protein that stabilizes the transition state.^30^ Molecular dynamics calculations on DS-epi1 with an octameric oligosaccharide indicated that the isomerized GlcA carboxylic functional group was often hydrogen bonded to Tyr261 and to protonated His205. In order to investigate the mechanism of isomerization of GlcA, a theozyme model of DS-epi1 was constructed from the core amino acids of isomerization, Tyr261, Tyr473 and His205, only including the C-alpha atoms from the protein backbone. A disaccharide from the reacting GlcA and the adjacent GalNAc in position –1 was also included in the theozyme model. All fragments were placed in accordance with a frame of a molecular dynamics trajectory which featured all the important hydrogen bonds, Tyr473 to Tyr261, Tyr261 to GlcA and protonated His205 to GlcA as well as a short distance from the hydrogen atom at the beta position of the carboxylate of GlcA to the oxygen of Tyr261.

With the intention of constraining the fragments to allowed positions in the full protein, locked coordinates were introduced for all C-alpha and C-beta atoms of the amino acids as well as for the two oxygens that formed glycosidic bonds to the deleted parts of the octamer. These rather rigid restrictions may cause high energies since both the protein and the carbohydrate are more flexible in the full protein than in the model. Additionally, in order for the isomerization of GlcA to occur, the His–GlcA hydrogen bond has to be charge separated to facilitate transfer of negative charge to Tyr261.

QM geometry optimizations of the theozyme with bound GalNAcβ1-4GlcA was performed for complex (S) which was modified in place to both the intermediary enol (I) and to the IdoA product (P). Both complexes (I and P) were subjected to QM geometry optimizations with the previously described constraints. A QM transition state search using the linear synchronous transit search method between the optimized (S) and (I) complexes was performed, resulting in transition state (T1, see ESI Video 2†). A corresponding transition state search was performed between the optimized (I) and (P1) complexes with a catalyzing water molecule added to both structures, resulting in transition state (T2). Vibrational analysis of (T1) and (T2) yielded only one large imaginary frequency for each, –1280.7 cm^–1^ and –1664.2 cm^–1^ respectively. For both transition states, animation of the imaginary frequency corresponded well to the reaction coordinate of the expected sigmatropic reaction.

### Proposed mechanism of DS-epi1

From the theozyme model we propose that the epimerization reaction starts by an electron shuttle through the carboxylic acid unit followed by proton abstraction from Tyr261 (Fig. 4B and C). The negative charge of the formed tyrosine anion is stabilized by a hydrogen bond from Tyr473 and provides a sufficiently strong base to abstract the acidic H5-proton of the GlcA unit in (T1). Proton abstraction from the charged His205 facilitates formation of a neutral enol (I). Both His205 and Tyr261 have previously been shown to be critical for catalytic activity.^19^ Mutagenesis of Tyr473 into an alanine similarly yielded an inactive protein (ESI Fig. 3†). In order to rule out that the differences in activity observed for the W98A and Y473A mutants were not due to protein misfolding, we performed structural modeling of the two mutants using a deep learning-based structure prediction implemented in trRosetta (ESI Fig. 4†).^31^ Only very small differences (RMSDs < 0.5 Å) were seen between the mutants and the wild-type protein, suggesting that the loss of activity was caused by disruption of catalytic properties and not incorrect folding. The top side of the enol is oriented towards the surrounding water layer and we propose that the enol can abstract a proton from one water molecule that in turn abstract a proton from the enol (T2), thus reforming the carboxylic acid unit (P). Alternatively, Asp147, located on the opposite side of the sugar plane, relative to Tyr473, might contribute to reprotonation. Mutation of Asp147 into an alanine reduced the activity by approximately 75% (ESI Fig. 3†).

### *N*-Glycosylation stabilizes the structure of DS-epi1

**Fig. 5:**
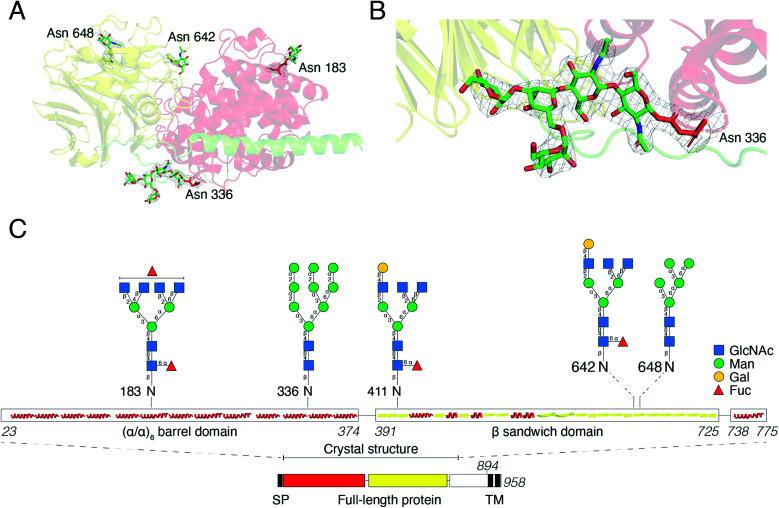
*N*-Glycosylation of DS-epi1. (A) Overview of *N*-glycans visible in the electron density from experimental data. The mesh map around the glycans is contoured at 1.5 sigma. (B) The glycan at Asn336 shows clear branching in the electron density, contoured at 1.5 sigma. (C) Cartoon showing the localization and structure of *N*-glycans identified by LC-MS/MS of DS-epi1-derived tryptic peptides. Images in (A) and (B) were prepared in PyMOL version 2.3.2.

For initial crystallization attempts, we expressed DS-epi1 in HEK293 cells which yielded natively glycosylated protein that crystallized and diffracted around 2.6 Å. To increase protein homogeneity and crystal quality, DS-epi1 was expressed in HEK293 GnTI(–) cells (devoid of *N*-acetylglucosaminyltransferase I (GnTI))^22^ which produce protein with only high-mannose type glycans. The high-mannose crystals diffracted at slightly higher resolution (2.4 Å) but no structural difference was observed between structures from the two datasets. For both structures, we found densities extending outwards from Asn 183, 336, 642 and 648, which could partly be modeled as *N*-glycans (Fig. 5A). The density at Asn336 revealed a clear sign of a branching glycan with two arms diverging from the third monosaccharide in a small raft between the alpha and beta domains. Five saccharide molecules were fitted in the electron density extending from ND2 of Asn336: Manα1–6(Manα1–3)Manβ1–4GlcNAcβ1–4GlcNAcβ1-Asn (Fig. 5B). In an attempt to even further improve the crystal packing, we attempted to express recombinant DS-epi1 in HEK293 GlycoDelete cells^32^ (which are GnTI(–) and engineered to express an endoT glycosidase in late Golgi) to produce protein with even more homogenous and truncated *N*-glycans. However, analysis of genomic DNA from the DS-epi1-expressing HEK293 GlycoDelete cells (after transfection and puromycin selection) revealed that the endoT gene, responsible for cleaving off high mannose type *N*-glycans in late Golgi in the engineered cell line, was missing (ESI Fig. 1H†).

We have previously characterized the *N*-glycosylation pattern of DS-epi1 by site-directed mutagenesis studies, where *N*-glycosylation was shown to be crucial for enzymatic activity.^19^ In those experiments, point mutations were introduced based on *in silico* predictions by the NetNGlyc web server, which identified four *N*-glycans at positions Asn 183, 336, 642 and 648. In order to yield more information about the glycan composition at each site, we performed an LC-MS/MS glycopeptide analysis of natively glycosylated full-length DS-epi1 from HEK293 cells. Glyco sites were identified based on observed fragment ions and calculated mass differences between non-modified and glycosylated peptides. In addition to glycosylations on the previously identified sites, we also observed a novel glycosite at Asn411. Both oligomannose and complex/hybrid structures were observed (Fig. 5C and ESI Table 2†) and a relatively high degree of glycan heterogeneity was observed for each site. The relative abundances of different glycoforms were calculated based on the observed extracted ion chromatograms and it was found that Asn 183, 336 and 642 were all fully glycosylated based on the absence of a non-glycosylated peptide. Only ∼2.5% of all identified Asn411-containing peptides were found to be glycosylated, explaining why the modification has not previously been discovered. Additionally, the unmodified peptide of Asn648 was too small to be detected by MS analysis, thereby disabling calculations of site occupancy. All glycopeptides from the Asn336 glyco site were found to be modified with high-mannose type glycans and the presence of high-mannose glycans was also confirmed by endoH degradation (ESI Fig. 1G†).

### The structure of full-length DS-epi1

**Fig. 6:**
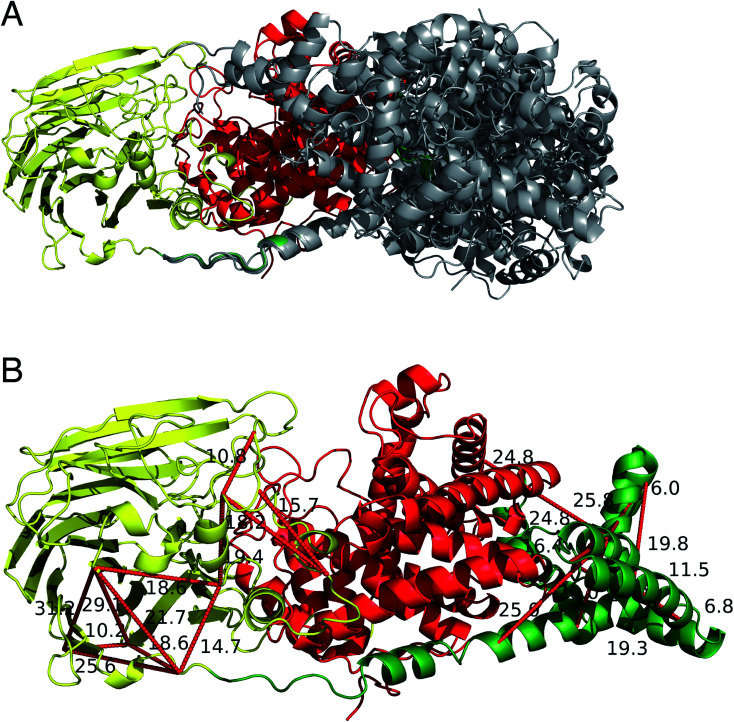
Extending the crystal structure of DS-epi1 by targeted cross-linking mass spectrometry (TX-MS). (A) Using the crystal structure as input to model the C-terminus, three thousand comparative models are provided by RosettaCM protocol to cover the conformational space of the calculations. The figure shows a subset of the highest scoring models superimposed. (B) Model of the highest combinatorial scoring of TX-MS and Rosetta energy function. Distance constraints from cross-linking mass spectrometry data were used by TX-MS to filter out the computational models and prepare the output, supported by 24 cross-links (visualized as red dotted lines, with Ångström distances annotated adjacently). Images were prepared in PyMOL version 2.3.2.

The two human dermatan sulfate epimerases, DS-epi1 (958 amino acids) and DS-epi2 (1212 amino acids), are predicted to be double-pass transmembrane proteins, with the active domains extending out into the Golgi lumen. They both share an N-terminal “epimerase” domain (51% identity between amino acids 43–673 and 62–691 for DS-epi1 and -2, respectively), while the C-termini bear no similarity to each other (ESI Fig. 5†). DS-epi2 contains a predicted sulfotransferase domain (amino acids 853–1202, Pfam PF00685) on the C-terminus, whereas no domain similarity to known structures has so far been reported for the last ∼280 amino acids of DS-epi1. Since the crystal structure of DS-epi1 does not include the full C-terminus of the protein, we aimed to extend the model to the complete luminal structure (amino acids 23–894) using cross-linking mass spectrometry. For this purpose, we used the targeted cross-linking mass spectrometry (TX-MS)^20^ with emphasis on intra cross-links to guide the structural modeling. Two different MS acquisition data were collected and analyzed; high resolution MS1 (hrMS1) and data dependent acquisition (DDA). The identified cross-links were used as experimental constraints to filter out the conformational space of generated models by Rosetta software suite.^33^ Accordingly, 24 intra cross-links were identified (ESI Fig. 6 and Table 3†). Rosetta *de novo*^34^ and RosettaCM^35^ protocols were used to explore the vast conformational space and represented several candidates for the C-terminus, which were then filtered out by the cross-linking experimental constraints (Fig. 6A). While 12 of these cross-links validated the crystal structure input, 12 were used to provide a model of the C-terminal domain (Fig. 6B). The C-terminal domain of all models that fulfilled the cross-link restraints (226 in total, ESI Table 4†) were compared to the full PDB (155625 models, downloaded on 2-Oct-19) using a local installation of DaliLite.v5,^36^ but no significant similarities was found for any model (ESI Table 5†), in agreement with primary sequence predictions. Finally, a hypothetical full-length protein including the transmembrane domain and complete *N*-glycans was constructed by glycan building, membrane construction/docking and energy minimization using the CHARMM web server (; http://www.charmm-gui.org) (ESI Fig. 7†).

### DS-epimerase expression among species and identification of bacterial homologs

To identify species with orthologs to human DS-epi1 and its only human paralog, DS-epi2, protein BLAST searches were performed against non-redundant protein sequence databases (GenBank CDS translations, PDB, SwissProt, PIR and PRF). The vast majority of DS-epi orthologs identified were predicted proteins based on automated computational analyses of genomic sequences, so all deviances where only one of the epimerases were identified in a species were carefully reviewed. The search identified 274 species expressing DS-epi1 and/or DS-epi2, all of which were identified to be metazoans (ESI Fig. 8, Tables 1 and 6†). Out of all species, *Chordata* was shown to represent the major phylum (98%, 264 species), with the remaining species belonging to the phyla *Hemichordata* (1), *Echinodermata* (3) and *Cnidaria* (6), all of which were unique for DS-epi2.

**Fig. 7:**
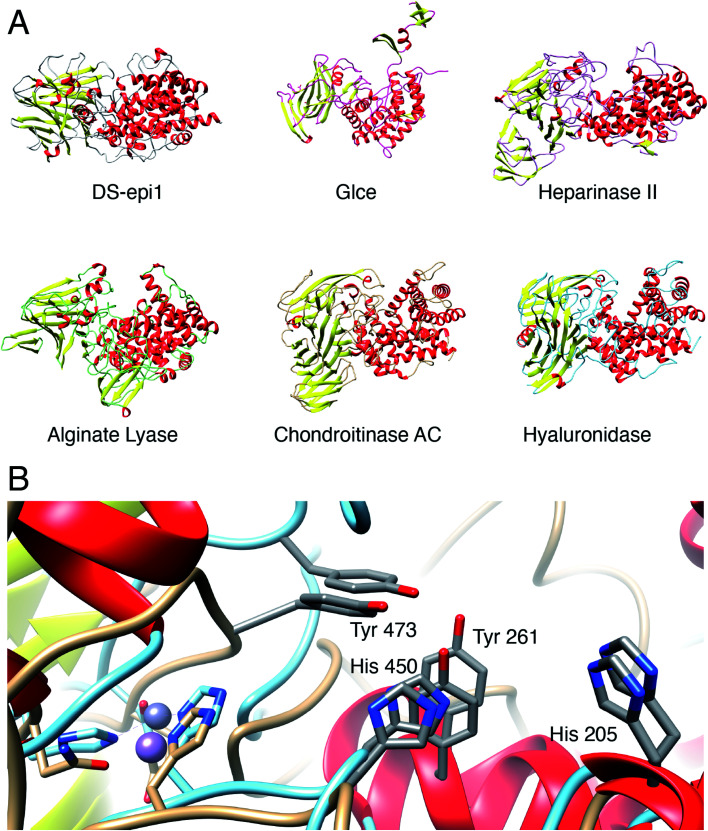
DS-epi1 is structurally similar to several bacterial polysaccharide lyases. (A) Several bacterial lyases belonging to polysaccharide lyase family 8 were found to possess overall structural similarity to DS-epi1. From top left to bottom right: DS-epi1 (*H. sapiens*), Glce (*H. sapiens*), heparinase II (*P. heparinus*), alginate lyase (*A. fabrum*), chondroitinase AC (*P. aurescens*), hyaluronate lyase (*S. pneumoniae*) (B) All catalytic amino acids of DS-epi1 (beige backbone), are conserved in the active site of heparinase II (turquoise backbone). In addition, both the metal-binding site and coordination geometry was conserved, where DS-epi1 coordinates a manganese ion and heparinase II a zinc ion.

In order to identify structural conservation of DS-epi1 among other proteins and species, we used the Dali server^37^ with the crystal structure of DS-epi1 as input. For all the structures in the PDB, DS-epi1 did not show any significant similarity to any eukaryotic protein (ESI Table 7†). Among the top hits when comparing the overall folds of the proteins were bacterial lyases belonging to polysaccharide lyase family 8, such as alginate lyase from *Agrobacterium fabrum* (PDB: ; 3A0O), heparinase II from *Pedobacter heparinus* (PDB: ; 2FUQ), chondroitinase AC from *Paenarthrobacter aurescens* (PDB: ; 1RWG), hyaluronate lyase from *Streptococcus pneumoniae* (PDB: ; 1W3Y) and heparinase III from *Pedobacter heparinus* (PDB: ; 4MMH) (Fig. 7A). Moreover, the N-terminal alpha helix domain of DS-epi1 was recognized as Pfam domain DUF4962, only found in dermatan-sulfate epimerases from mammals and polysaccharide lyases from Gram-negative bacteria. Several of the active site histidines and tyrosine were conserved in the lyases. For example, in heparinase II, distinct similarities were found both in the active site, but also in the metal binding site (Fig. 7B). None of the bacterial proteins could be identified when using only the primary sequence of DS-epi1. We also performed BLAST searches using the sequence of DS-epi2 from more primitive non-chordates, but no further proteins were identified.

## Discussion

An experimentally supported model of the full-length Golgi luminal DS-epi1 was created, where 80% of the 958 amino acid-protein was solved by macromolecular crystallography and the rest using targeted cross-linking mass spectrometry (TX-MS) experiments and Rosetta modeling. The combination of macromolecular crystallography and TX-MS provides a method to solve complete structures of larger proteins where only part of the structure can be solved by crystallography experiments. At the same time, it is useful to confirm similarities between protein conformations in solution and crystal structures, which may give new information of kinetic conformational changes. The demonstration that TX-MS experiments in combination with macromolecular crystallography are feasible for structural studies is important as this approach can also be used in studies of interactions of other biosynthetic proteins and complex organizers in cells. The full-length model revealed a three-domain protein where the first two domains, the α and β domains, harbor the main enzymatic function. The third, C-terminal, domain is of unknown function. However, we have previously described that at least ∼60 of the first amino acids of the domain need to be included in recombinant constructs in order to yield an enzymatically active protein.^21^ The crystallographic structure reveals that the first part of the domain consists of a ∼30 amino acid long alpha helix which connects and possibly stabilizes the α and β domains. The structure of the C-terminal domain is most likely unique to DS-epi1 since no similar domains could be identified neither based on the primary sequence nor on the structural level. One possible function of the C-terminal domain is to enable homo- and heteromerization of the protein, which has previously been observed in FRET experiments and co-immunoprecipitations.^23^ However, since no dimerization was observed in SEC experiments with the full-length protein, it might be that an additional protein is necessary for dimerization, or that the Golgi micromilieu is significantly different from the *in vitro* experiments we have performed.

Musculocontractural Ehlers–Danlos syndrome (mcEDS) is a rare disease caused by mutations in DS-epi1 or the IdoA-specific enzyme dermatan 4-*O*-sulfotransferase 1. To this date, only eight mcEDS patients with mutations in the DSE gene have been described in the literature.^10^ In order to investigate the potential impact of the mutations on the protein structure, we looked closer at the three patients carrying missense mutations (excluding truncating and frameshift variants). Two of the mutations, Arg267Gly^9^ and Ser268Leu,^8^ are located centrally in helix α9 of the (α/α)_6_-toroid domain, whereas the third, His588Arg,^11^ is found in the β10 strand of the β-supersandwich domain, at a distance to the active site of approximately 15 and 20 Å for Arg267/Ser268 and His588, respectively (ESI Fig. 5†). Both locations are in line with previous reports showing that a large proportion of disease-associated mutations are found buried in protein cores, where they may cause destabilization of the protein structure.^38^ When looking at the electron density, it is clear that the *Nτ* of His588 hydrogen bonds to the backbone-carbonyl oxygen of Phe479. However, no clear amino acid interactions could be seen for Arg267 or Ser268. In the original reports describing the mcEDS mutations Arg267Gly and Ser268Leu, the authors describe marked reduction of IdoA-containing disaccharides in the affected patients, but not complete absence. Based on published results from the DS-epi1 and DS-epi1/2 knockout mice, it is likely that the residual IdoA-residues are the product of DS-epi2 activity.^39^,^40^ However, it cannot be excluded that some activity still remains in the DS-epi1 mutants.

In this study, we propose a new catalytic mechanism of DS-epi1, involving the catalytic triad His205, Tyr261 and Tyr473. Both new and previous experiments, *in vitro* and *in vivo*, have confirmed one or more of the suggested catalytic amino acids to be crucial for activity, which is further supported by a deeply conserved core including the catalytic site, identified in the crystal structure.^19^,^41^ Heparan sulfate (HS) is also modified by a GlcA-converting epimerase on the polymer level. In contrast to the two human DS-epimerases, only one human HS epimerase (Glce) exists, acting on substrates containing *N*-sulfated hexosamine residues. Crystal structures of both the zebrafish and the human Glce protein have been solved and the protein has been shown to exist as a dimer containing two catalytic sites.^42^,^43^ Each monomeric unit consists of three domains: an N-terminal β-hairpin domain, a central β-sandwich domain and a C-terminal (α/α)_4_-barrel domain. The substrate binding pocket is situated in a positively charged (at pH 7) cleft made up mainly of the (α/α)_4_-barrel domain. No similarities can be identified on the primary sequence level between Glce and DS-epi1, but structurally, the C-terminal (α/α)_4_-barrel and central β-sandwich domains of Glce resembles truncated forms of the N-terminal (α/α)_6_-toroid and central β-supersandwich domains of DS-epi1. However, the N-terminal β-hairpin domain of Glce is missing in DS-epi1, the C-terminal domain of DS-epi1 is not present in Glce and unlike DS-epi1, the metal binding site (Ca^2+^) is located far away from the active site in Glce. Additionally, the double tyrosine motif is located opposite to the β-sandwich domain in the substrate-binding groove, contrary to the location in DS-epi1; the pH optima are different (∼7 for Glce and ∼5.5 for DS-epi1 (ref. 26)); although *N*-glycosylated, the number and distribution of glycans is different for Glce. Still, the overall shape, as well as the general organization of the active site amino acids, of DS-epimerase 1 is similar to the human Glce (Fig. 7A), and an analogous arrangement of two tyrosine units of Glce, *i.e.* Y578 and Y560, indicates a similar mechanism. The same arrangement is also found in heparinase II (PDB: ; 2FUQ) and alginate lyase (PDB: ; 3A0O). We propose that this double tyrosine motif is a general feature that may be found in other lyases and epimerases, and that minute changes in the active sites can give significant changes in enzyme activity, *i.e.* from a lyase to an epimerase.

In close vicinity to the active site, His452, Glu470 and Asn481 coordinates a manganese ion. Mutagenesis experiments revealed that all three amino acids are crucial for catalytic activity and/or proper protein folding (for Asn481). His452 is located on the same loop as His450 which interacts with the carboxylate moiety of GlcA. Conformational changes of this loop will likely have an impact on the catalytic activity, suggesting that manganese coordination is directly involved in the catalytic properties of the enzyme and not only for conformational integrity. Regarding substrate binding, the entire active site cleft was found to shift from a negative to a positive surface potential as the pH decreased from 7–6, similar to the pH optimum of DS-epi1 and the pH found in the Golgi lumen, which likely is a requirement for binding of the negatively charged polysaccharide substrate.^44^ We also identified a tryptophan residue, Trp98, lined up in the active site, which could contribute to carbohydrate–aromatic interactions with the pyranose rings of the substrate, in agreement with previous reports on the interaction modes of enzymes acting on neutral polysaccharides.^45^ We have previously shown that DS-epi1 works in a processive way, resulting in the generation of several adjacent IdoA-GalNAc disaccharides.^21^ Further experiments are needed in order to investigate the specific mechanism underlying the stepwise epimerizations, also taking into consideration the *in vivo* micro milieu created by the interaction of DS-epi1 and sulfotransferases.^23^

More than 50% of all human proteins are thought to be glycosylated^46^ and aberrant glycosylation patterns are known to affect both folding and function of proteins. In contrast, only ∼5% of all human protein structures in the PDB database contain glycans.^47^ Most post-translational protein modifications introduce microheterogeneities resulting in surface variations between the molecules. In addition, the relatively long and flexible glycan entities may substantially increase the entropy at the surface. The resulting loss of structurally homogeneous molecules are well known reasons for severely hampering the crystallization process or decrease the quality of crystals. However, on a practical level, glycosylations are in many cases well accommodated by water channels in the crystals or constitute parts of crystal contacts, and crystallography experiments with glycosylated proteins many times have comparable success rates to non-glycosylated proteins.^48^ For DS-epi1, it was possible to achieve diffraction-quality crystals using a fully glycosylated protein. In addition, several glycans were also clearly visible in the electron density. By extending the information from the crystal structure with mass spectrometric glycopeptide analysis of wild-type HEK293 protein, we generated a more complete picture of the *N*-glycosylation of DS-epi1 and extended the known glycosylation pattern to include a partly *N*-glycosylated site on Asn411. From previous experiments, we have shown that full glycosylation of the epimerase is required for activity.^19^ The high-mannose glycan on Asn336 is positioned parallel to the linker peptide connecting the α and β domains, where it may play an important role for the interaction and stability of the two domains. This is also supported by the fact that only Asn 336 and 648 are conserved in DS-epi2 (ESI Fig. 5†) and that DS-epi1 could not be expressed in GlycoDelete cells which can only produce *N*-glycan stubs of sialylated trisaccharides.

Both DS-epi1 and DS-epi2 were found to be broadly distributed in *Chordata*. However, in simpler organisms such as *Cnidaria*, *Echinodermata* and *Hemichordata*, only DS-epi2 was found, suggesting that DS-epi2 is the older of the two human DS-epimerases. The consequence of the observed difference in DS-epi expression in more primitive organisms should be investigated. As for the structure of DS-epi1, it reveals a high structural similarity to proteins from several families of bacterial polysaccharide lyases (*e.g.* CAZy PL8, PL12 and PL21). Since the products of epimerization (and subsequent sulfation) are substrates for several of the bacterial lyases, it is tempting to believe that the ancestral gene was of eukaryotic origin. Further, GAGs are not found in more basal metazoans (*e.g. Porifera*), which suggests that the enzymes are the result of a horizontal gene transfer event.^49^ However, even though horizontal gene transfers have been extensively described, transfers from eukaryotes to bacteria is less common than *vice versa*.^50^ It remains to be understood whether a gene transfer event has occurred, or if all genes have evolved from a common ancestral gene.

In summary, we present the first structure of human *N*-glycosylated human dermatan sulfate epimerase 1, expressed in mammalian cells. The unique structure of the enzyme, only found in metazoans, highlights the importance of C5-epimerization of uronic acid in higher organisms. The structure will be essential for generation of inhibitors, which may function as drugs for cancer and fibrosis.^16^,^51^,^52^

## Materials and methods

### Expression and purification of DS-epi1

DS-epi1 23–775 and 23–894 was cloned and expressed as previously described, with the following modifications: DS-epi1 23–775 used for crystallization was expressed in the HEK293 GnTI-cell line (a kind gift from Professor Nico Callewaert, Ghent University, Belgium) which produces protein with a homogenous *N*-glycosylation pattern composed of high-mannose-type glycans.^21^,^53^ Size-exclusion chromatography was performed on a Superose 200 increase 10/300 mm column (GE Healthcare Life Sciences) using a running buffer composed of HEPES (20 mM, pH 7.9), NaCl (150 mM), and MnCl_2_ (2 mM). The column was operated at 0.5 ml min^–1^ and monomeric fractions were pooled and concentrated using 30 kDa MWCO Amicon Ultra centrifugal concentrators (Millipore). The general yield of pure protein was in the range of 1–4 mg per liter culture.

### Crystallization and data collection

The purified DS-epi1 at a concentration of 7.3 mg ml^–1^ in buffer containing 20 mM HEPES pH 7.9, 150 mM NaCl, 2 mM MnCl_2_ used for setting up drops using commercially available screens. An initial crystal hit was obtained by the sitting drop vapor diffusion method with a protein to reservoir volume ratio of 200 : 200 nl and incubated with a 45 ml reservoir at 20 °C in a triple drop UV polymer plate (Molecular Dimensions, UK). A mosquito nanoliter pipetting robot (TTP Labtech, UK) was used to set up drops, which were imaged by the Minstrel HT UV imaging system (Rigaku Corporation, USA) available at the Lund Protein Production Platform (LP3), Lund University. Crystals were obtained with a reservoir containing 200 mM NH_4_CH_3_CO_2_, 100 mM MES pH 6.5 and glycerol ethoxylate (15/4 EO/OH) 30% v/v (condition #G7 of the molecular dimensions MIDAS screen). The crystals were then further optimized by hanging drop using a Nextal plate and diffraction quality crystals were obtained within 1 week from a crystallization solution with 6% xylitol as an additive. Crystals were picked up directly from the drop where glycerol ethoxylate present in the crystallization solution worked as cryoprotectant. Anomalous data were collected at 100 K using the ID29 beamline at the ESRF,^54^ Grenoble, France and native data collected at BioMAX beamline^73^ at MAX IV laboratory, Lund, Sweden. The crystals are in space group *C*222(1) with unit cell dimensions *a* = 182.68 Å, *b* = 213.91 Å, *c* = 86.95 Å, *α* = 90.0, *β* = 90.0, *γ* = 90.0, containing one molecule of DS-epi1 in the unit cell. The solvent content is 72.12%.

### Structure determination and model building

The diffraction images were integrated using XDS^55^ and scaled using Aimless^56^ from the CCP4 package.^57^ The structure was solved by anomalous phasing using the Crank2 package,^58^ where 6 anomalous sites from manganese ions were found by SHELX^59^ and substructure was determined by SHELXD.^60^ Density modification was done by Solomon,^61^ Multicomb^62^ and Parrot.^63^ Model extension by Buccaneer^64^ and refinement by Refmac5.^65^ The Crank2 package could build a partial model with 604 residues with a final *R*/*R*-free of 42.71%/46.66%. The resulted model was then used in Phaser^66^ to calculate a density map with a higher resolution data set with a TFZ score of 33.6 and LLG of 1217. The resulting map was corrected and extended using Phenix Autobuild wizard.^67^ This obtained structure was rebuilt and corrected manually in repeated cycles of Coot^68^ and Buccaneer, then refined to convergence using phenix.refine.^69^ The quality of the refined structure of glycans were checked with Privateer.^70^ Data collection and refinement statistics are found in Table 1.

### Molecular dynamics simulations

Molecular dynamics simulations were performed with the OPLS3 force field in Desmond implemented in Schrödinger Release 2020-1 using default settings except for the length of the simulation and the use of light harmonic constraints (1 kcal mol^–1^ Å^–2^) on all stranded and helix backbone atoms.

### DFT calculations

QM calculations were performed with Jaguar implemented in Schrödinger Release 2020-1. Gas phase geometries were optimized at the M06-2X/6-31g** level of theory with D3 *a posteriori* corrected dispersion. The PBF solvation model with water solvent was then applied through single point energy calculations at the same level of theory.

### Construction of plasmids for expression of DS-epi1 point mutants

Point mutations were introduced into the pCEP-Pu-DS-epi1_23–775_ plasmid by PCR amplification using a Platinum SuperFi II polymerase (Thermo Fisher Scientific) and the primers in Table 2.

**Table 2:** Sequences of primers used to create DS-epi1 point mutants. Lowercase letter indicates site of mutation

Name	Sequence
W98A-F	TTCTGCCAGAgccAATGAGATTTTCGG
W98A-R	TAATCCTTTGGGTCCCAAG
D147A-F	TGCCCCATGGgccGAGGTGCCCC
D147A-R	TCTTTCACCAGCCATGAGGGCTGTGC
N204A-F	GTATCTGCACgccCATCAGCCCACAAAC
N204A-R	TGAAATCCCCACCCTCTC
F319A-F	CTACAATTGGgccTATGGGCCCGAG
F319A-R	TTACTGTCTGCGATAGCC
P383A-F	AAGTGTGCCAgccCCTGACTTCG
P383A-R	TTCAGAGAGCCATCGTACCAC
H452A-F	TGGACACGAAgccCCTGACCAGAATTCC
H452A-R	GCATTGAAGTTGCGCCAC
E470A-F	CTTCATCACTgccGCCCTGTACGGC
E470A-R	GGCACTCCGTTAGGTGCA
Y473A-F	TGAGGCCCTGgccGGCCCAAAGT
Y473A-R	GTGATGAAGGGCACTCCG
N481A-F	TACCTTCTTTgccAATGTGCTGATGTTCTC
N481A-R	TACTTTGGGCCGTACAGG

PCR-amplified products were phosphorylated with T4 PNK (NEB), ligated using Quick T4 DNA ligase (NEB) and then used to transform DH5-alpha competent *E. coli* (Thermo Fisher Scientific). Plasmids were purified using PureLink fast low-endotoxin midi plasmid purification kit (Thermo Fisher Scientific) and finally sequenced (Eurofins Genomics).

### Expression of DS-epi1 point mutants

Expi293F suspension cells (Thermo Fisher Scientific) were cultured in Expi293 expression medium (Thermo Fisher Scientific) at 37 °C in an 8% CO_2_ incubator, with shaking at 130 rpm (19 mm orbit). Cells, at a density of 3 × 10^6^ cells per ml (>95% viability), were transfected with mutant plasmids using 1 μg DNA per ml culture and 3 μl polyethyleneimine “MAX 40K” (Polysciences, Inc.) per μg DNA. After 20 h, sodium valproate and anti-clumping supplement (Irvine Scientific, 91150) were added to 3 mM and 2 ml l^–1^, respectively. The cell culture supernatant was harvested after 72 hours and clarified by centrifugation (2000 g for 10 min) and filtration (0.45 μm PES filter unit, Thermo Fisher Scientific).

### Western blot analysis

Conditioned culture medium was mixed with Laemmli buffer and proteins were separated on a 4–15% Mini-PROTEAN TGX stain-free protein gel (Bio-Rad) using a tris-glycine-SDS buffer (Bio-Rad). Proteins were then transferred to PVDF membranes, which were subsequently blocked (EveryBlot, Bio-Rad) and then probed with a home-made rabbit anti-DS-epi1 (1 μg ml^–1^, antigen: amino acids 509–520) in blocking buffer (EveryBlot, Bio-Rad). A goat anti-rabbit IgG (H + L)-HRP conjugate (Bio-Rad, 1706515) was used together with a clarity western ECL substrate (Bio-Rad) to develop the blot, which was imaged using a ChemiDoc imaging system (Bio-Rad).

### Epimerase activity analysis

Epimerase activity was measured as previously described, with slight modifications.^5^ Conditioned culture medium was dialyzed against a buffer consisting of 20 mM MES (pH 5.5 at 37 °C), 10% glycerol, 0.5 mM EDTA, 0.1% Triton X-100 and 1 mM dithiothreitol. Culture medium from cells transfected with an empty vector was included as negative control and medium from cells transfected with a plasmid encoding wild-type DS-epi1 23–775 was used as a positive control. The desalted medium samples (80 μl each) were mixed with a substrate cocktail (20 μl) containing 0.1 mg of bovine serum albumin, 2 mM MnCl_2_, 0.5% Nonidet P-40, and 30 000 dpm [5-^3^H]-chondroitin. After 18 h at 37 °C, the sample was boiled and subsequently centrifuged at 20 000 g for 5 min. The supernatant was distilled and tritium release was quantified using a Hidex 600SL automatic TDCR liquid scintillation counter.

### Size-exclusion chromatography and molecular weight determination

To compare the mono- or multimeric state of DS-epi1 at different protein concentrations, proteins were separated on an AdvanceBio SEC UHPLC column (300 Å, 2.7 μm, 4.6 × 300 mm, Agilent) with a mobile phase consisting of HEPES (20 mM), NaCl (150 mM) and MnCl_2_ (5 mM).

For absolute molecular weight determination, multi-detection SEC was performed using a Malvern Panalytical OMNISEC system (Malvern, UK) consisting of Refractive Index (RI), right angle and low angle light scattering (RALS/LALS) and differential viscometer. For chromatographic separation, a Superdex 200 increase 10/300 (GE Life Sciences) was used with a mobile phase consisting of HEPES (20 mM) and NaCl (150 mM). A d*n*/d*c* of 0.185 ml g^–1^ was used to process the protein samples. All data was collected and processed using OMNISEC v10.

### Protein digestion of glycopeptides for MS analysis

Full-length DS-epi1 (amino acids 23–894, without signal peptide and transmembrane regions) with native glycosylation was dissolved in 50 mM triethylammonium bicarbonate (TEAB) to give protein concentrations of 1 μg μl^–1^. Based on protein sequence evaluation, chymotrypsin was selected as proteolytic enzyme providing the best access to all potential *N*-glycosylation sites. Protein samples (20 μg) were reduced with 4.1 mM dithiothreitol (DTT) at 60 °C for 30 min and alkylated with 8.3 mM 2-iodoacetamide (IAM) for 30 min at room temperature (in the dark), following additional 15 min incubation with 4.1 mM DTT to react with excess of IAM prior to the chymotrypsin digest. Chymotryptic digest was performed overnight at 37 °C by addition of Pierce™ MS grade chymotrypsin (0.25 μg, Thermo Fisher Scientific). Digestion was stopped by acidification with 10% trifluoroacetic acid and samples were desalted using Pierce™ peptide desalting spin columns (Thermo Fischer Scientific) following the manufacturer's guidelines. The salt free supernatants were dried down and reconstituted in 2% acetonitrile (ACN) in 0.1% formic acid (FA) for LC-MS analysis.

### NanoLC MS analysis of glycopeptides

Digested samples were analyzed on a Q Exactive HF mass spectrometer interfaced with Easy-nLC1200 liquid chromatography system (Thermo Fisher Scientific). Peptides were trapped on an Acclaim Pepmap 100 C18 trap column (100 μm × 2 cm, particle size 5 μm, Thermo Fischer Scientific) and separated on an in-house packed analytical column (75 μm × 300 mm, particle size 3 μm, Reprosil-Pur C18, Dr Maisch) using a gradient from 7% to 50% B over 75 min, followed by an increase to 100% B for 5 min at a flow of 300 nl min^–1^, where solvent A was 0.2% FA and solvent B was 80% ACN in 0.2% FA. The instrument operated in data-dependent mode where the precursor ion mass spectra were acquired at a resolution of 120 000, *m*/*z* range 600–2000. The 10 most intense ions with charge states 2 to 5 were selected for fragmentation using HCD at collision energy settings of 28. The isolation window was set to 3 *m*/*z* and dynamic exclusion to 20 s. MS2 spectra were recorded at a resolution of 30 000 with maximum injection time set to 110 ms.

### *N*-Glycan database search

For sample and digest quality control, the acquired data were first analyzed using Proteome Discoverer version 1.4 (Thermo Fisher Scientific). A database search was performed with the Mascot search engine, version 2.5.1 (Matrix Science) using the *Homo sapiens* Swissprot database with 20 244 sequences with precursor mass tolerance of 5 ppm and fragment mass tolerance of 200 millimass units. Variable modification of methionine oxidation and fixed cysteine alkylation were selected. Chymotryptic peptides with up to 6 missed cleavage were accepted. The detected peptide threshold in the software was set to a significance level of Mascot 95% by searching against a reversed database and identified proteins were grouped by sharing the same sequences to minimize redundancy. This analysis confirmed the successful isolation and sufficient proteolytic digestion of the target protein, dermatan-sulfate epimerase 1 (DS-epi1).

The Byonic software (Protein Metrics) was used to identify glycopeptides from two different DS-epi1 preparations. The precursor and fragment ion tolerance were set to 10 ppm and 20 ppm, respectively. Fully specific cleavage after FWYL with up to 5 missed cleavages were accepted. The “309 mammalian *N*-glycans” database generated by the Consortium for Functional Glycomics (; https://www.functionalglycomics.org) together with Met oxidation were allowed as variable modifications. Cysteine carbamidomethylation was set as static modification. Byonic's glycopeptide identifications were manually evaluated prior to the final assignment of the observed glycosylation forms for each glycopeptide. The extracted ion chromatograms (EIC) of the identified glycopeptides were used to determine site-specific glycoform distributions (microheterogeneity). For each observed glycoform, an average intensity was calculated from the EIC peak intensities of three independent injections. For each *N*-glycosylation site, all glycosylated and non-glycosylated peptides sharing the same chymotryptic peptide sequence were used for evaluation of the site microheterogeneity. The relative abundance of each glycoform was calculated as percent of the summed intensities of all detected glycoforms for the given glycosylation site.

### Sample preparation for targeted cross-linking mass spectrometry (TX-MS)

DS-epi1 23–894 (full-length, minus transmembrane domains) was used as input material for the TX-MS experiments. Six micrograms of DS-epi1 was resuspended in HEPES buffer (20 mM, pH 7.0), supplemented with MnCl_2_ (15 mM), at 37 °C, 800 rpm for 30 min. Heavy/light disuccinimidyl suberate (DSS-H12/D12, Creative Molecules Inc.) resuspended in dimethyl sulfoxide (DMSO) was added to final concentrations of 0.5 mM and incubated for an additional 30 min at 37 °C, 800 rpm. The reaction was quenched with a final concentration of 50 mM Tris (pH 7.5) at 37 °C, 800 rpm, for 15 min. Cross-linked samples were denatured in urea (6 M in 100 mM NH_4_HCO_3_) and reduced with a final concentration of 6.7 mM tris(2-carboxyethyl)phosphine at 37 °C, 800 rpm, for 45 min. Reduced cysteines were alkylated with iodoacetamide (final concentration 6.7 mM) at 22 °C for 30 min. Samples were diluted to 1.5 M urea in 100 mM NH_4_HCO_3_ and sequencing-grade lysyl endopeptidase (1.25 μg, 37 °C, 2 h) (Wako Chemicals) followed by trypsin (2 μg, 37 °C, 18 h) (Promega) was added to digest the protein into peptides. The peptide-containing samples were acidified with formic acid to pH 3.0 and the peptides were purified by C18 reversed-phase columns according to the manufacturers protocol (Macro SpinColumns Silica C18, Harvard Apparatus). Purified peptides were dried and reconstituted in 2% acetonitrile supplemented with 0.2% formic acid.

### LC-MS/MS of TX-MS samples

All peptide analyses were performed on a Q Exactive Plus mass spectrometer (Thermo Scientific) connected to an EASY-nLC 1000 ultra-high-performance liquid chromatography system (Thermo Scientific) essentially as described^20^ with a few modifications. For data dependent analysis (DDA), peptides were separated on an EASY-Spray column (Thermo Scientific; ID 75 μm × 50 cm, column temperature 45 °C) operated at a constant pressure of 600 bar. A linear gradient from 3% to 35% acetonitrile in aqueous 0.1% formic acid was run for 60 min at a flow rate of 300 nl min^–1^. One full MS scan (resolution 70 000 @ 200 *m*/*z*; mass range 400–1600 *m*/*z*) was followed by MS/MS scans (resolution 17 500 @ 200 *m*/*z*) of the 15 most abundant ion signals. The precursor ions were isolated with 2 *m*/*z* isolation width and fragmented using higher-energy collisional-induced dissociation at a normalized collision energy of 30. Charge state screening was enabled, and precursors with an unknown charge state and singly charged ions were rejected. The dynamic exclusion window was set to 10 s. The automatic gain control was set to 1 × 10^6^ for both MS and MS/MS with ion accumulation times of 100 and 60 ms, respectively. The intensity threshold for precursor ion selection was set to 1.7 × 10^4^. For high-resolution MS1 (hrMS1), peptides were separated using an EASY-Spray column (Thermo Scientific; ID 75 μm × 50 cm, column temperature 45 °C) operated at a constant pressure of 600 bar as in DDA and DIA. A linear gradient from 3% to 35% acetonitrile in aqueous 0.1% formic acid was run for 60 min at a flow rate of 300 nl min^–1^. High-resolution MS scans (resolution 280 000; mass range 400–2000 *m*/*z*) were acquired using automatic gain control (AGC) set to 3 × 10^6^ and a fill time of 200 ms.

### Structural modeling of the DS-epi1 C-terminus using the TX-MS protocol

To provide the structural inputs for the modeling, three separate steps were taken each of which was guided by constraints derived from cross-linking MS. First, Rosetta *de novo* modeling protocol was used to model the C-terminus of DS-epi1 by producing 3000 models. Next, the small transmembrane domain was modeled using Rosetta comparative modeling protocol (RosettaCM) by producing 200 models. For the final structure, 3000 models were provided using RosettaCM protocol by incorporating the top models from the previous steps. All structural models were filtered by cross-linking data as the experimental constraints using TX-MS approach, yielding a final 226 models (ESI Table 4†).^20^ Finally, two different scoring systems have been combined to select the final model. First, the Rosetta energy function is used to select the top 20 models and then, a normal distribution score based on cross-link length is considered in a way, cross-links with length between 15 Å and 25 Å obtained better score in comparison to shorter or longer cross-links with respect to the main threshold of 32 Å.

### Dali protein structure comparisons

All Rosetta models were compared to the complete PDB (155625 models, downloaded on 2/10–19) using a cluster installation of DaliLite.v5.^36^ The highest scoring hit for each model is summarized in ESI Table 5.†

### *In silico* exploration of species expressing DS-epimerases

A manually curated protein BLAST search was performed against the non-redundant protein databases (GenPept, Refseq, PDB, SwissProt, PIR and PRF). For DS-epi1, part of the DS-epi1-specific human C-terminal domain, ranging from amino acid 755–898, was used as query sequence. For DS-epi2, amino acids 731–1010, part of the predicted human DS-epi2-specific *O*-sulfotransferase domain, was used as query. All predicted splice forms were ignored and only one corresponding ortholog for DS-epi1 and -2 was selected for each species.

Alignments for phylogenetic analyses were prepared using Clustal Omega.^71^ A circular phylogenetic tree, rooted in *Homo sapiens*, was constructed using Interactive Tree Of Life (iTOL) version 4.^72^ Taxonomy IDs were assigned using Batch Entrez (; https://www.ncbi.nlm.nih.gov/sites/batchentrez) and leaf labels were automatically added based on taxonomy ID.

### Graphical representations

All graphical representations were prepared in UCSF Chimera version 1.13.1 (build 41965), unless otherwise stated.

### Data availability

Coordinates and structure factors for DS-epi1 have been deposited in the protein data bank under accession code 6HZN. Other data are available from the corresponding author upon request.

## Author contributions

ET, AM, UE, GWT and UM designed and coordinated the study. ET, MH, HK, LH, AS and JU performed the experiments and analyzed the data. ET, MH, HK, LH, JU, JM, LM, UE and AM interpreted and reviewed the results. ET, MH, HK, UE and AM wrote the article, which was reviewed and approved by all of the authors.

## Conflicts of interest

The authors declare no conflicts of interest.

## Supplementary Material

Supplementary information

Supplementary movie

Supplementary movie

Supplementary information

Supplementary information

Supplementary information

Supplementary information

Supplementary information

Supplementary information

Supplementary information

Supplementary information
