# Supplementary material for: The structure of human dermatan sulfate epimerase 1 emphasizes the importance of C5-epimerization of glucuronic acid in higher organisms
Source: Chem Sci. 2020 Dec 8;12(5):1869–85. doi: 10.1039/d0sc05971d (PMC8006597; doi:10.1039/d0sc05971d)
Supplement: Supplementary file 4 [file SC-012-D0SC05971D-s004.pdf]

Electronic Supplementary Material (ESI) for Chemical Science.
This journal is © The Royal Society of Chemistry 2020

Supplementary figures

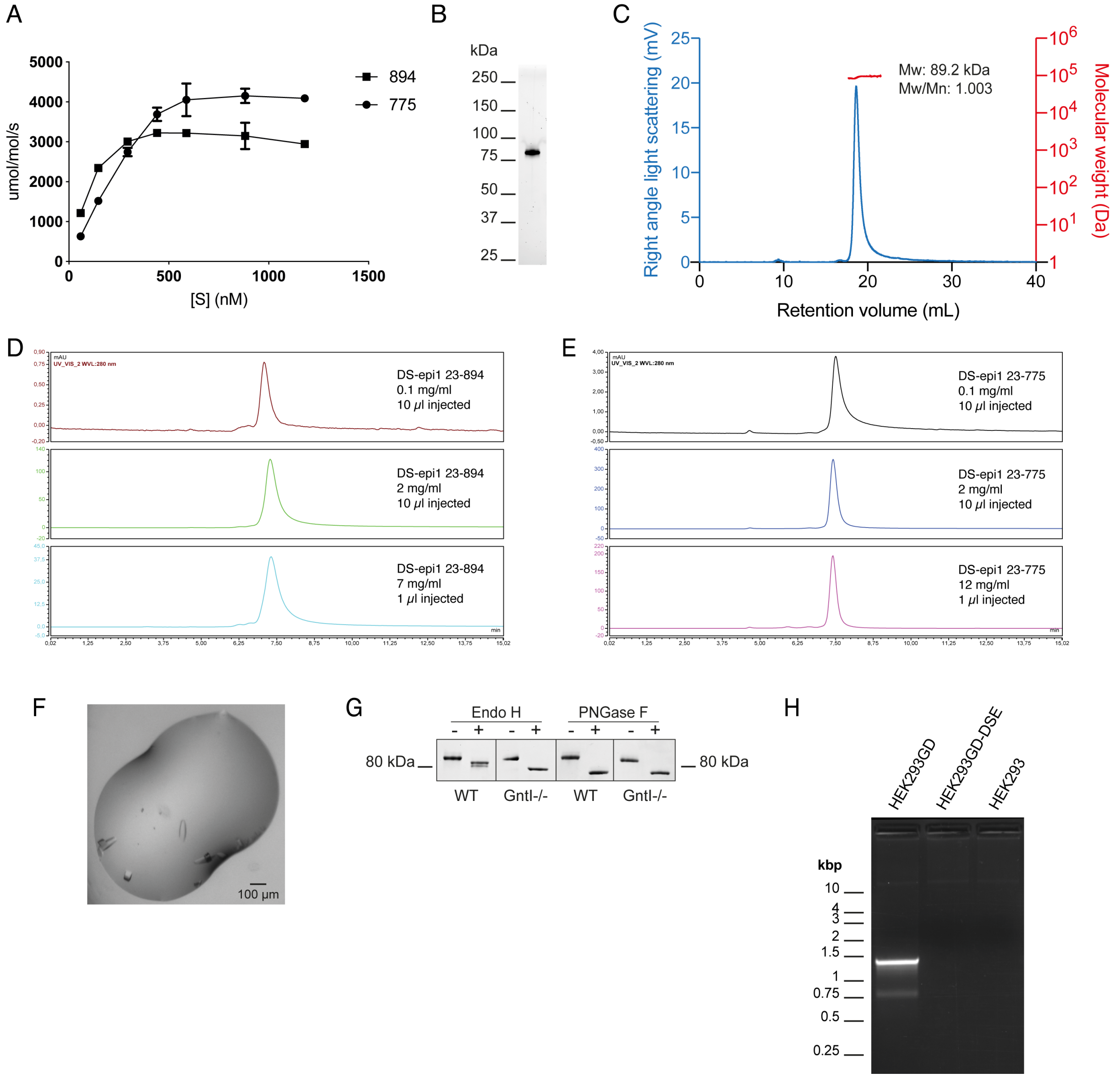

Fig. S1. Biochemical characterization of recombinant DS-epi1.

(A) Enzymatic activity of DS-epi1 23-894 and 23-775, using C5-tritiated chondroitin as substrate. Each (B) SDS-PAGE analysis (C) Multi-angle light scattering analysis, revealing a monomeric and monodisperse product of 89.2 kDa (theoretical Mw=87.2 kDa). (D) and (E) size-exclusion chromatography (SEC) analysis of DS-epi1 23-894 and 23-775 at different protein concentrations. Proteins were separated on an AdvanceBio SEC UHPLC column. (F) Crystals from a drop containing 200 mM $NH_4CH_3CO_2$, 100 mM MES pH 6.5, 30% v/v glycerol ethoxylate (15/4 EO/OH) and 6% xylitol (G) Glycosidase degradation and SDS-PAGE analysis (H) PCR analysis of the bacterial endoT gene using genomic DNA.

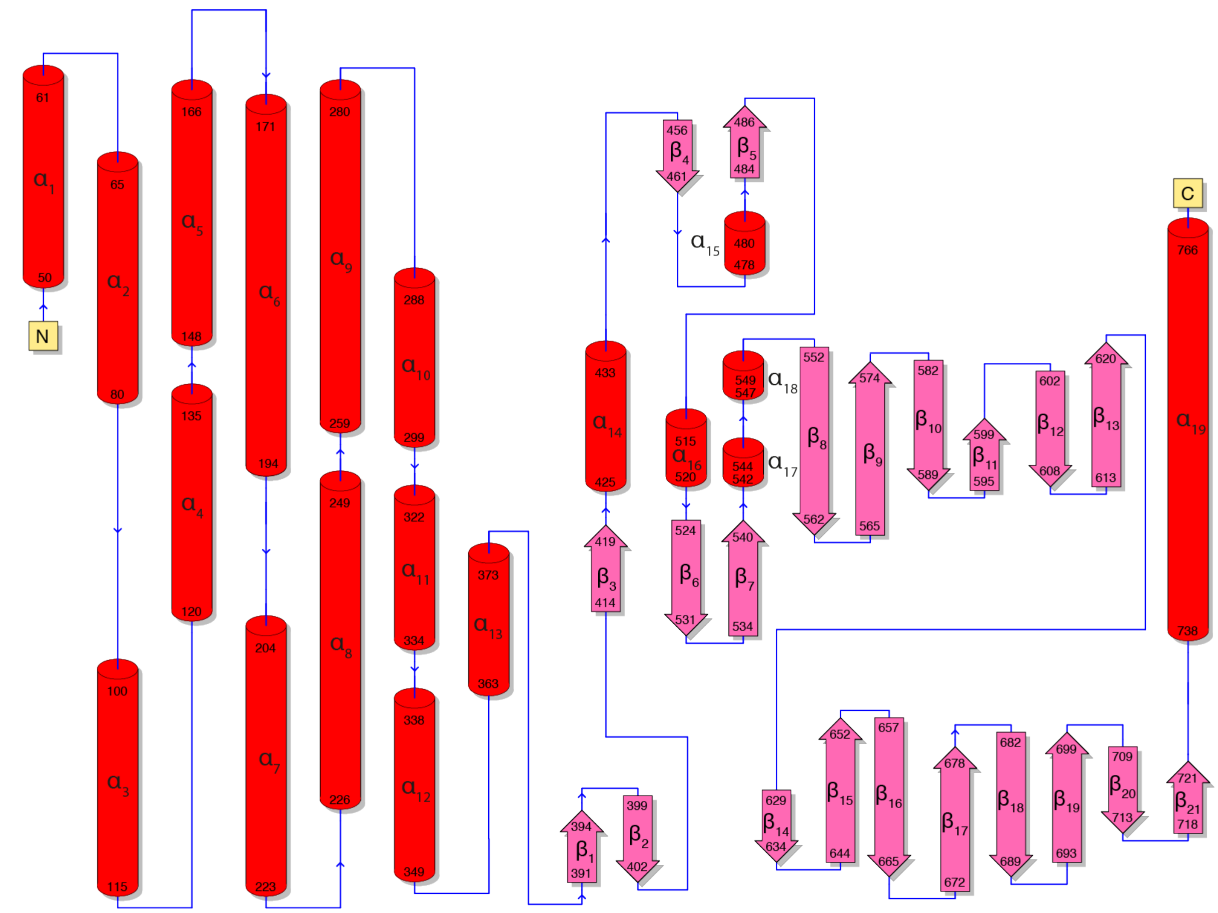

Fig. S2. Topology scheme of the crystal structure of DS-epi1. Alpha helices are shown as cylinders and labeled $\alpha_{1\text{-}20}$. Beta strands are shown as arrows and labeled $\beta_{1\text{-}21}$. Each corresponding starting and end amino acid is shown for each element and the N- and C-termini are marked with N and C. The scheme, based on HERA, was generated using PDBsum and further modified manually for clarity reasons.

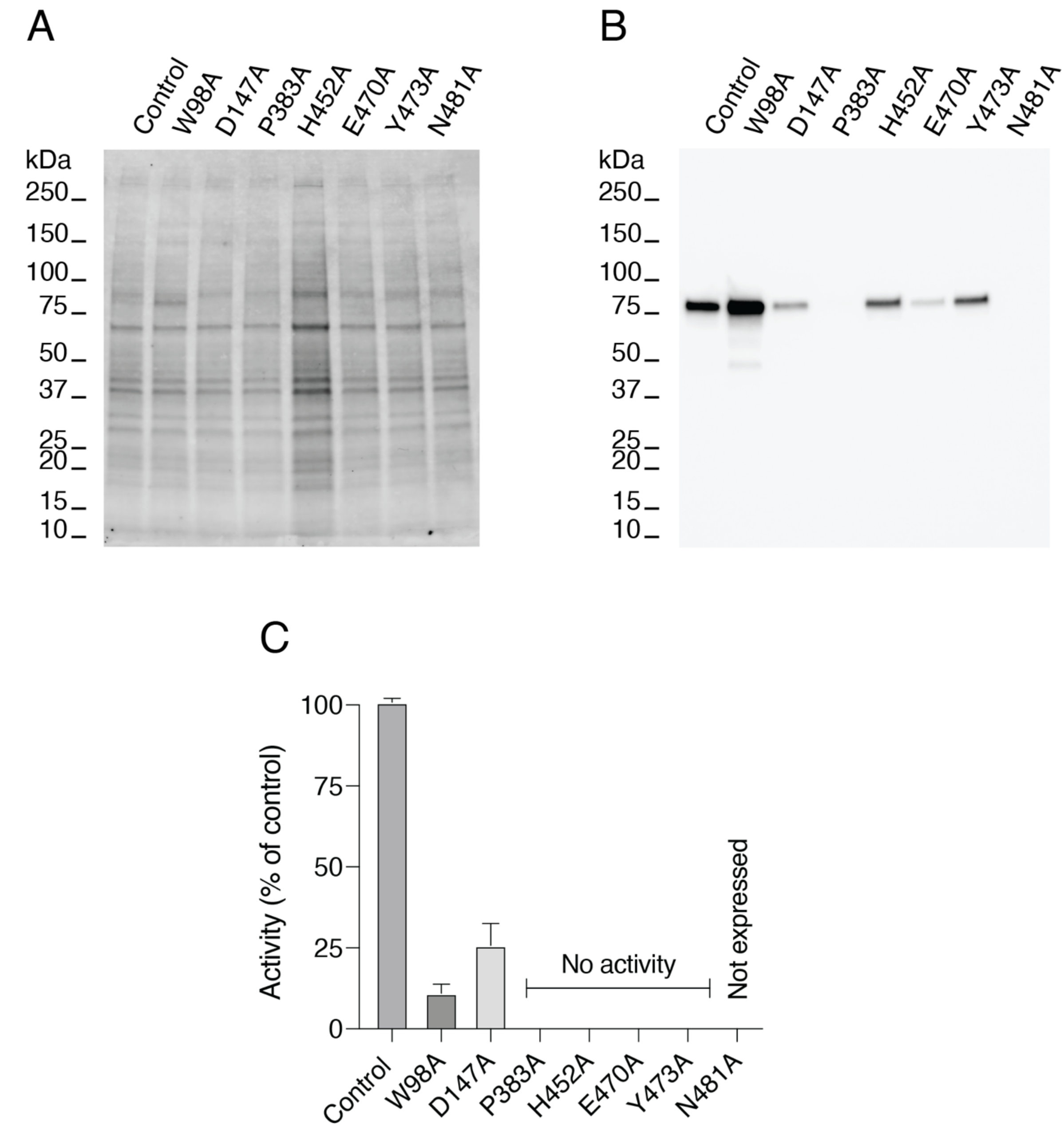

Fig. S3. Western blot and activity analysis of DS-epi1 23-775 point mutants. (A) Culture supernatants from HEK293 cells transfected with wild-type control plasmid or plasmids with point mutants were separated by SDS-PAGE and transferred to a PVDF membrane. Image shows total protein visualized using Bio-Rad stain-free imaging technology. (B) PVDF membrane probed with primary rabbit anti-DS-epi1 antibody and secondary anti-rabbit HRP conjugate, and developed using a chemiluminescent HRP substrate. (C) Activity analysis of DS-epi1 products using a C5-tritiated chondroitin substrate, followed by distillation and liquid scintillation counting. The activity of each product was normalized to the expression, as analyzed by western blot, and expressed as % of DS-epi1 23-775 wild-type control. The P383A product was barely visible after development of the western blot membrane, whereas the N481A product could not be detected. Each experiment was performed in duplicate and expressed as mean with standard deviation.

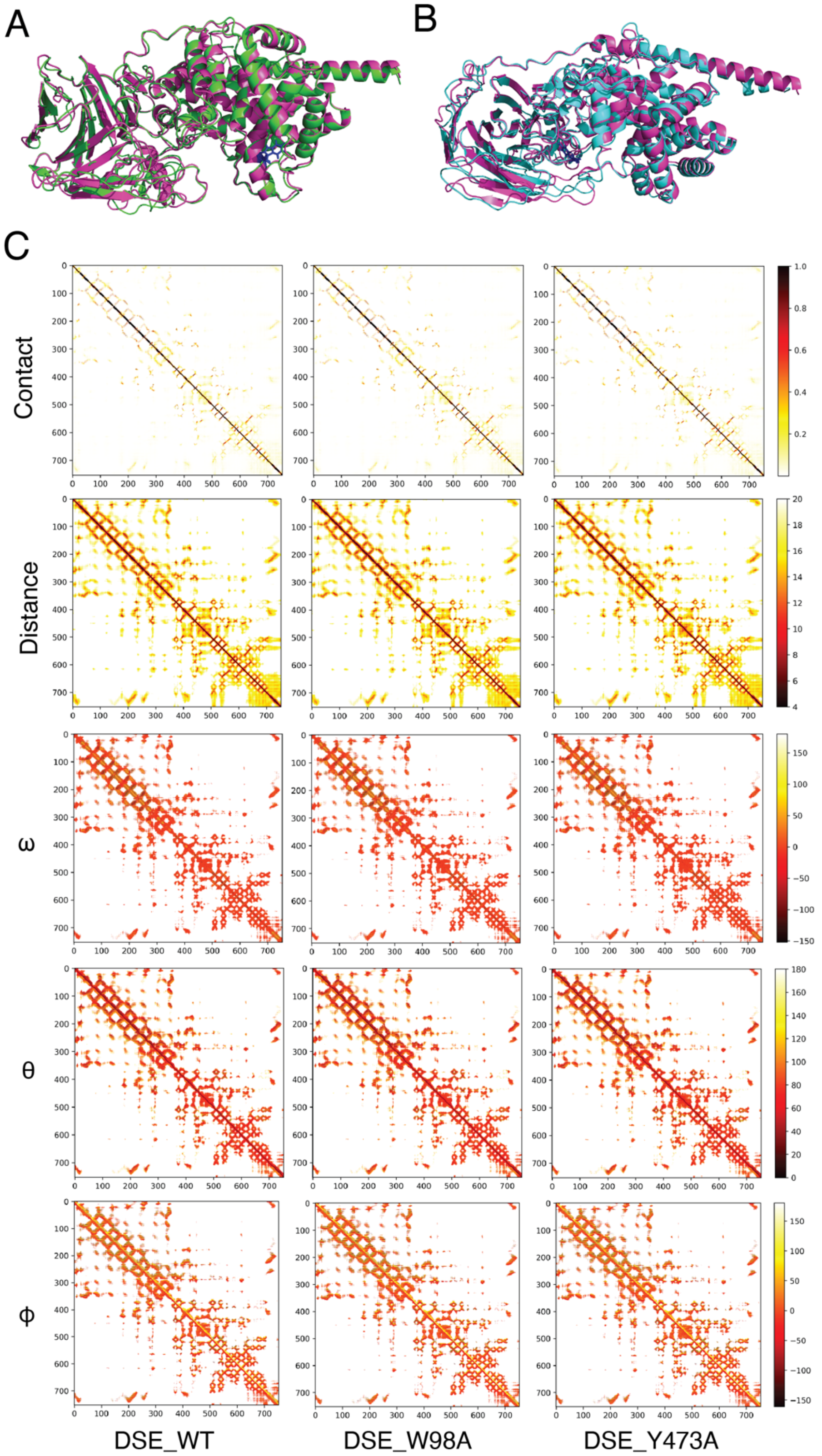

Fig. S4. DS-epi1 wild-type and mutant structure prediction using trRosetta (https://yanglab.nankai.edu.cn/trRosetta/). Structure predictions are based on a deep residual network for predicting inter-residue orientations and distances, followed by a Rosetta-constrained energy-minimization protocol to generate structure models. (A) Structural alignment between DS-epi1 WT (purple) and W98A mutant (green), with an RMSD of 0.43 Å (B) Structural alignment between DS-epi1 WT (purple) and Y473A mutant (turquoise), with an RMSD of 0.45 Å (C) Predicted inter-residue contacts/distances/orientations for DS-epi1 wild-type, W98A and Y473A. Residue number on x and y axis.

```
DS-epi1, human (Uniprot ID Q9UL01)
DS-epi2, human (Uniprot ID Q8IZU8)

1      MRTHTRGAPSVFFIYLLCFVSAYITDE--NPEVMIPFTNANYDS--------------------HPMLYFSRAEVAELQL  58
1      MALMFTG--HLLFLALLMFAFSTFEESVSNYSEWAVFTD-DIDQFKTQKVQDFRPNQKLKKSMLHPSLYFDAGEIQAMRQ  77

                                                    Trp 98
59     RAASSHEHIAARLTEAVHTMLSSPLEYLPPWDPKDYSARWNEIFGNNLGALAMFCVLYPENIEARDMAKDYMERMAAQPS  138
78     KSRASHLHLFRAIRSAVTVMLSNPTYYLPPPKHADFAAKWNEIYGNNLPPLALYCLLCPEDKVAFEFVLEYMDRMVGYKD  157

              Asp 147                                    Asn 183               Asn 204, His 205
139    WLVKDAPWDEVPLAHSLVGFATAYDFLYNYLSKTQQEKFLEVIANASGYMYETSYRRGWGFQYLHNHQPTNCMALLTGSL  218
158    WLVENAPGDEVPIGHSLTGFATAFDFLYNLLDNHRRQKYLEKIWVITEEMYEYSKVRSWGKQLLHNHQATNMIALLTGAL  237

                                                       Tyr 261  Arg 267 + Ser 268
219    VL-MNQGYLQEAYLWTKQVLTIMEKSLVLLREVTDGSLYEGVAYGSYTTRSLFQYMFLVQRHFNINHFGHPWLKQHFAFM  297
238    VTGVDKG--SKANIWKQAVVDVMEKTMFLLNHIVDGSLDEGVAYGSYTAKSVTQYVFLAQRHFNINNLDNNWLKMHFWFY  315

                                              Asn 336
298    YRTILPGFQRTVAIADSNYNWFYGPESQLVFLDKFVMRNGSGNWLADQIRRNRVVEGPGTPSKGQRWCTLHTEFLWYDGS  377
316    YATLLPGFQRTVGIADSNYNWFYGPESQLVFLDKFILKNGAGNWLAQQIRKHRPKDGPMVPSTAQRWSTLHTEYIWYDPQ  395

                                   Asn 411                                    His 450+452
378    LKSVPPPDFGTPTLHYFEDWGVVTYGSALPAEINRSFLSFKSGKLGGRAIYDIVHRNKYKDWIKGWRNFNAGHEHPDQNS  457
396    LTPQPPADYGTAKIHTFPNWGVVTYGAGLPNTQTNTFVSFKSGKLGGRAVYDIVHFQPYS-WIDGWRSFNPGHEHPDQNS  474

           Glu 470   Tyr 473   Asn 481
458    FTFAPNGVPFITEALYGPKYTFFNNVLMFSPAVSKSCFSPWVGQVTEDCSSKWSKYKHDLAASCQGRVVAAEEKNGVVFI  537
475    FTFAPNGQVFVSEALYGPKLSHLNNVLVFAPSPSSQCNKPWEGQLGE--CAQWLKWTGEEVGDAAGEIITASQHGEMVFV  552

                                                                His 588
538    RGEGVGAYNPQLNLKNVQRNLILLHPQLLLLVDQIHLGEESPLETAASFFHNVDVPFEET---VVDGVHGAFIRQRDGLY  614
553    SGEAVSAYSSAMRLKSVYRALLLLNSQTLLVVDHIERQEDSPINSVSAFFHNLDIDFKYIPYKFMNRYNGAMMDVWDAHY  632

                                Asn 642       Asn 648
615    KMYWMDDTGYSEKATFASVTYPRGYPYNGTNYVNVTMHLRSPITRAAYLFIGPSIDVQSFTVHGDSQ---QLDVFIATSK  691
633    KMFWFDHHGNSPMASIQEAEQAAEFKKRWTQFVNVTFQMEPTITRIAYVFYGPYINVSSCRFIDSSNPGLQISLNVNNTE  712

692    HA------YATYLWTGEATGQSAFAQVIADRHKILFDRNSAIKSSIVPEVKDYAAIVEQNLQHFKPVFQLLEKQILSRV-  764
713    HVVSIVTDYHNLKTRFNYLGFGGFASV-ADQGQIT--RFGLGTQAIVKPVRHDRIIFPFGFKFNIAVGLILCISLVILTF  789

765    --RNTASFRKTAERLLRFSDKRQTEEAIDRIFAISQ-------QQQQQSKSKKNRRAGKRYKFVDAV--------PDIFA  827
790    QWRFYLSFRKLMRWILILVIALWFIELLDVWSTCSQPICAKWTRTEAEGSKKSLSSEGHHMDLPDVVITSLPGSGAEILK  869

828    QIEVNEKKIRQKAQILAQKELPIDEDEEMKDLLDFADVTYEKHKNGG--LIKGRFGQARMVTTTHSRAPSLSASYTRLFL  905
870    QLFFNSSDFLYIRVPTAYIDIP-ETELEIDSFVDACEWKVSDIRSGHFRLLRGWLQSLVQDTKLHLQNIHLHEPNRG---  945

906    ILNIAIFFVMLAMQLTYFQRAQSLHGQRC------------------------------------------LYAVLLIDS  943
946    --KLAQYFAMNKDKKRKFKRRESLPEQRSQMKGAFDRDAEYIRALRRHLVYYPSARPVLSLSSGSWTLKLHFFQEVLGAS  1023

944    CILL--------WLYSSCSQSQC----------------------------------------------------------  958
1024   MRALYIVRDPRAWIYSMLYNSKPSLYSLKNVPEHLAKLFKIEGGKGKCNLNSGYAFEYEPLRKELSKSKSNAVSLLSHLW  1103

    --------------------------------------------------------------------------------
1104   LANTAAALRINTDLLPTSYQLVKFEDIVHFPQKTTERIFAFLGIPLSPASLNQILFATSTNLFYLPYEGEISPTNTNVWK  1183

       -----------------------------
1184   QNLPRDEIKLIENICWTLMDRLGYPKFMD  1212
```

Fig. S5. Sequence alignment between human DS-epimerases. Sequences were aligned using COBALT Constraint-based Multiple Alignment Tool (https://www.ncbi.nlm.nih.gov/tools/cobalt/cobalt.cgi). Proposed active-site residues are marked with black rectangles, glycosylation sites are marked with brown rectangles, metal-coordinating sites are marked with blue rectangles and mutations linked to musculocontractural Ehlers-Danlos syndrome are marked with red rectangles.

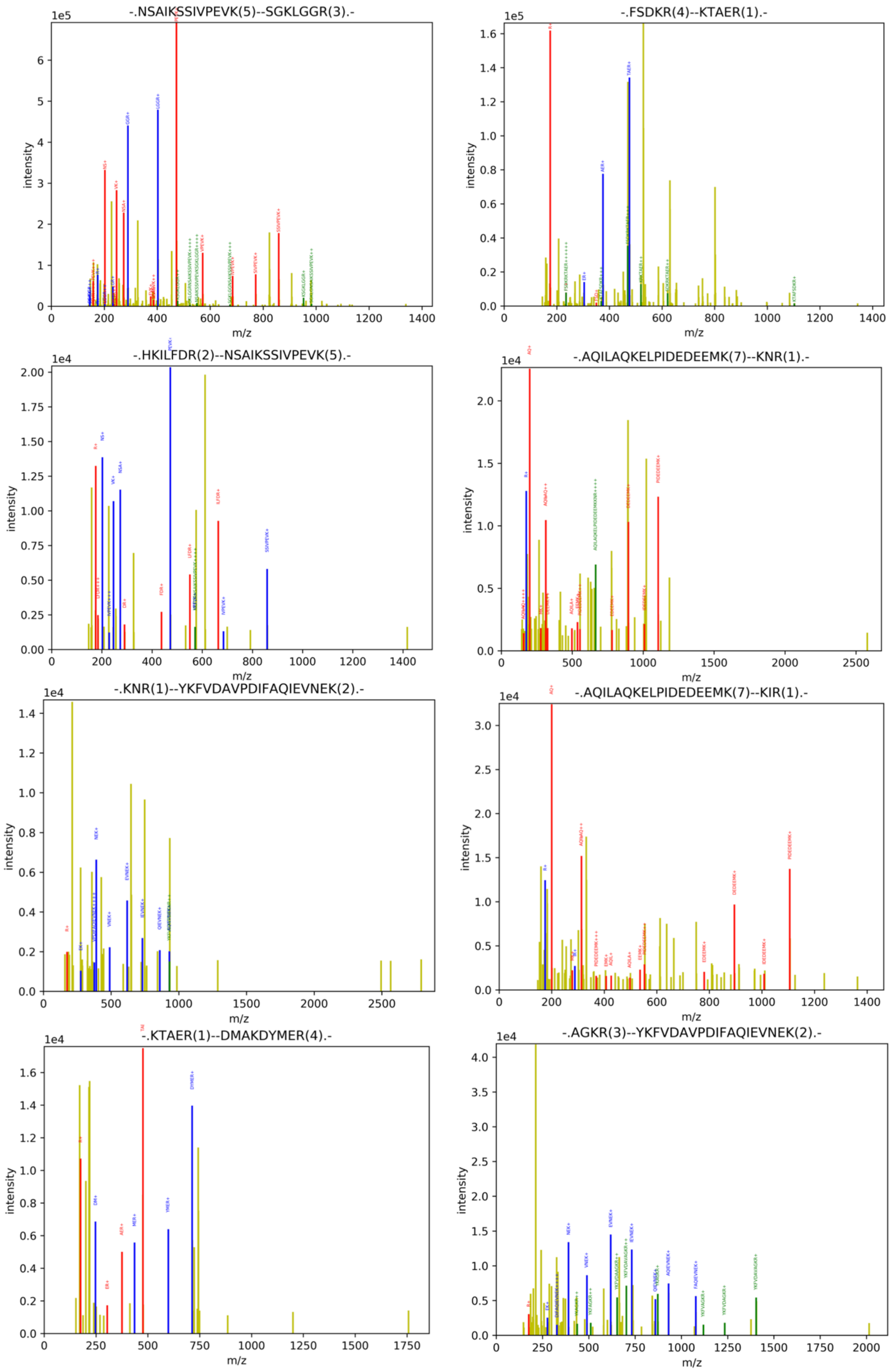

Fig. S6A. Supporting spectra for TX-MS table S2. In each spectrum figure, the title refers to the reference XL peptides. The yellow color shows the background spectrum which is found in the MS/MS sample. The red, blue, and green colors are corresponding to the first peptide fragments, second peptide fragments, and both peptides with cross-linking agent (DSS), respectively.

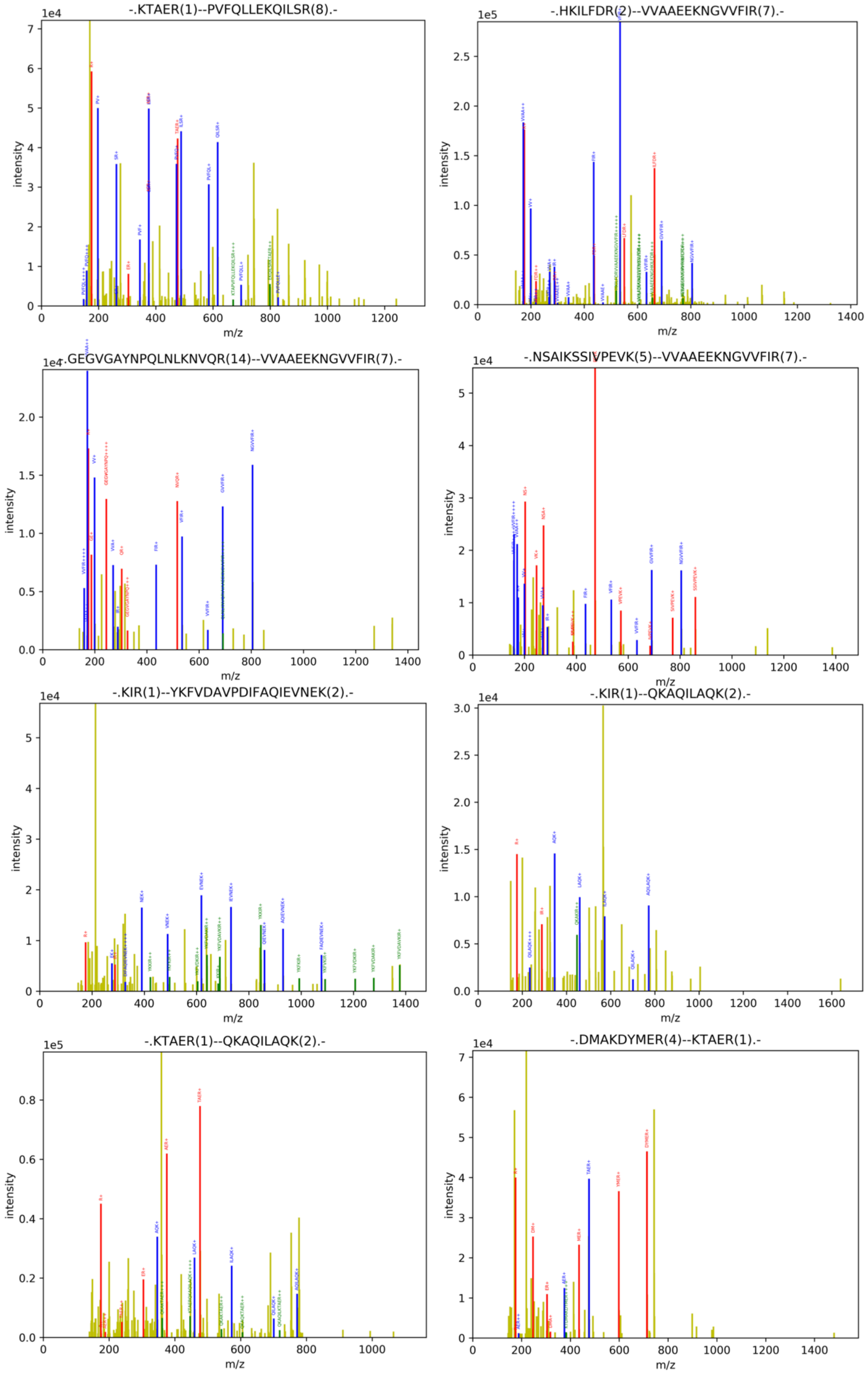

Fig. S6B. Supporting spectra for TX-MS table S2. In each spectrum figure, the title refers to the reference XL peptides. The yellow color shows the background spectrum which is found in the MS/MS sample. The red, blue, and green colors are corresponding to the first peptide fragments, second peptide fragments, and both peptides with cross-linking agent (DSS), respectively.

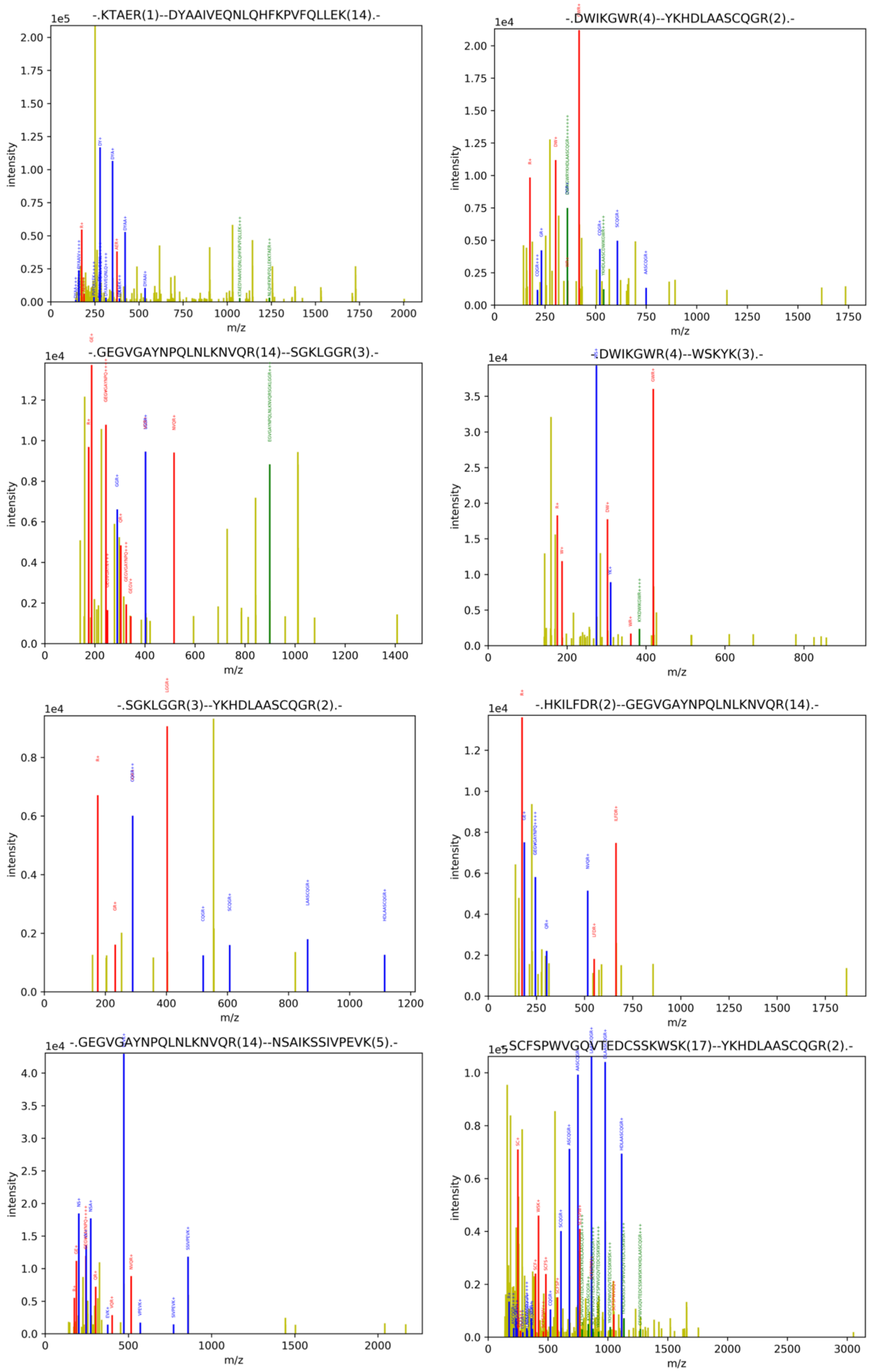

Fig. S6C. Supporting spectra for TX-MS table S2. In each spectrum figure, the title refers to the reference XL peptides. The yellow color shows the background spectrum which is found in the MS/MS sample. The red, blue, and green colors are corresponding to the first peptide fragments, second peptide fragments, and both peptides with cross-linking agent (DSS), respectively.

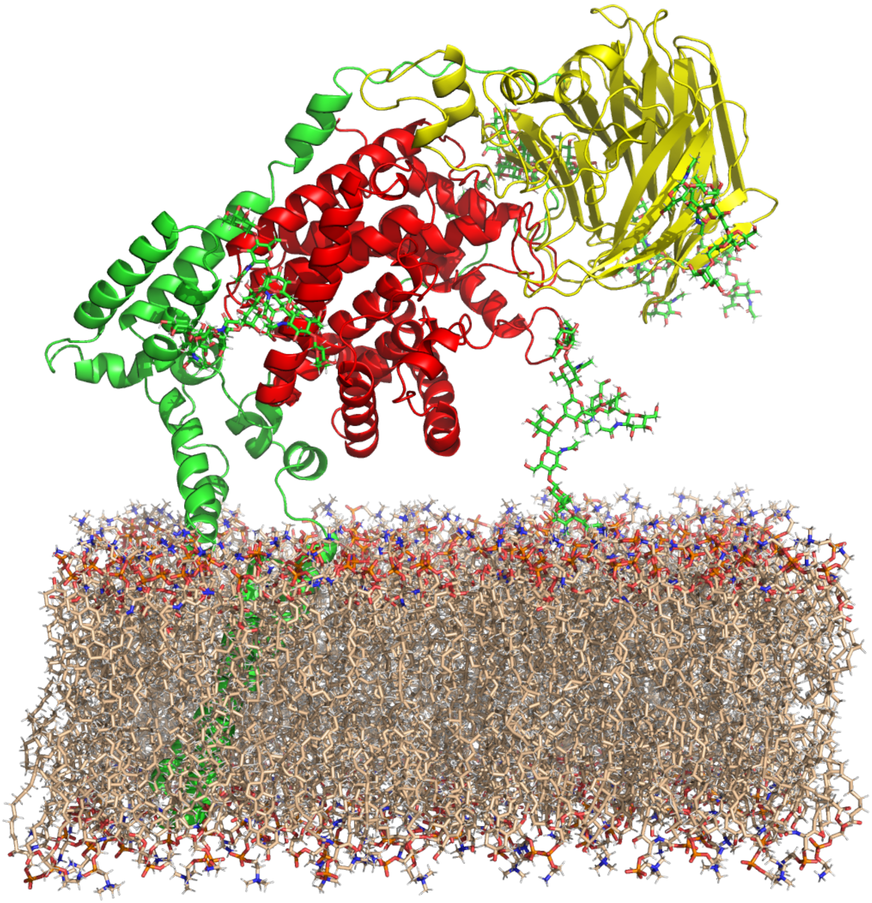

Figure S7. Model of full-length human DS-epi1. The model was constructed by glycan building, membrane construction/docking and energy minimization using the CHARMM web server (http://www.charmm-gui.org). The modeled glycans and C-terminal domain are shown in green.

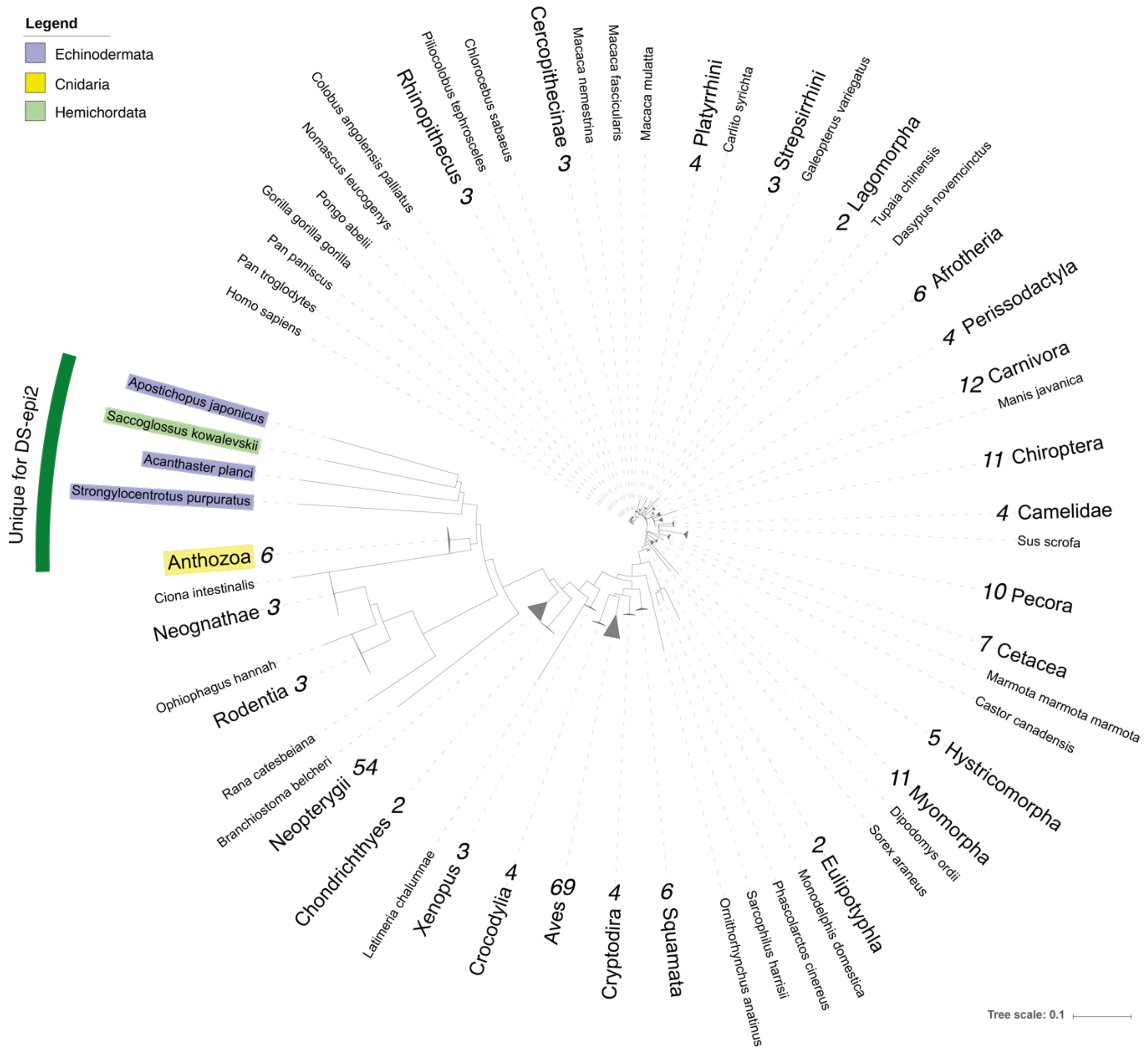

Fig. S8. Phylogenetic tree of species expressing DS-epi1 and/or DS-epi2, rooted in homo sapiens. All species identified were animals and the majority chordates. Non-chordates, all unique for DS-epi2, are colored according to the legend on the top left. Nodes with multiple species belonging to the same class, order or family were collapsed and the numbers adjacent to those nodes correspond to the number of species in each node. The isosceles triangle for each collapsed node is proportional to the number of species in the node.
