# Supplementary material for: The structure of human dermatan sulfate epimerase 1 emphasizes the importance of C5-epimerization of glucuronic acid in higher organisms
Source: Chem Sci. 2020 Dec 8;12(5):1869–85. doi: 10.1039/d0sc05971d (PMC8006597; doi:10.1039/d0sc05971d)
Supplement: Supplementary file 7 [file SC-012-D0SC05971D-s007.pdf]

Electronic Supplementary Material (ESI) for Chemical Science.
This journal is © The Royal Society of Chemistry 2020

**Supplementary Table 3. List of cross-links (XL) constraints found by analyzing mass spectrometry data.** All 24 XLs support the final output structure with Euclidean distance shown in the last column. The distance cut-off is set to 32 Å.

| XL (kojak format) | Lysine 1 position | Lysine 2 position | Euclidean distance (Å) |
|---|---|---|---|
| *Intra β-sandwich domain* | | | |
| -.NSAIKSSIVPEVK(5)--SGKLGGR(3).- | 728 | 421 | 15.32 |
| -.HKILFDR(2)--NSAIKSSIVPEVK(5).- | 718 | 728 | 26.07 |
| -.HKILFDR(2)--VVAAEEKNGVVFIR(7).- | 718 | 531 | 10.41 |
| -.NSAIKSSIVPEVK(5)--VVAAEEKNGVVFIR(7).- | 728 | 531 | 18.12 |
| -.GEGVGAYNPQLNLKNVQR(14)--SGKLGGR(3).- | 552 | 421 | 20.74 |
| -.SGKLGGR(3)--YKHDLAASCQGR(2).- | 421 | 514 | 20.91 |
| -.DWIKGWR(4)--YKHDLAASCQGR(2).- | 441 | 514 | 19.32 |
| -.DWIKGWR(4)--WSKYK(3).- | 441 | 512 | 15.54 |
| -.HKILFDR(2)--GEGVGAYNPQLNLKNVQR(14).- | 718 | 552 | 31.14 |
| -.GEGVGAYNPQLNLKNVQR(14)--NSAIKSSIVPEVK(5).- | 552 | 728 | 23.60 |
| -.SCFSPWVGQVTEDCSSKWSK(17)--YKHDLAASCQGR(2).- | 509 | 514 | 9.25 |
| -.GEGVGAYNPQLNLKNVQR(14)--VVAAEEKNGVVFIR(7).- | 552 | 531 | 28.24 |
| *Intra C-terminal domain* | | | |
| -.KIR(1)--YKFVDAVPDIFAQIEVNEK(2).- | 835 | 817 | 18.11 |
| -.KIR(1)--QKAQILAQK(2).- | 835 | 839 | 5.94 |
| -.KTAER(1)--QKAQILAQK(2).- | 772 | 839 | 23.42 |
| -.KTAER(1)--DYAAIVEQNLQHFKPVFQLLEK(14).- | 772 | 750 | 26.13 |
| -.KTAER(1)--PVFQLLEKQILSR(8).- | 772 | 758 | 17.90 |
| -.AGKR(3)--YKFVDAVPDIFAQIEVNEK(2).- | 814 | 817 | 9.40 |
| -.AQILAQKELPIDEDEEMK(7)--KIR(1).- | 846 | 835 | 20.51 |
| -.KNR(1)--YKFVDAVPDIFAQIEVNEK(2).- | 808 | 817 | 11.87 |
| -.AQILAQKELPIDEDEEMK(7)--KNR(1).- | 846 | 808 | 31.86 |
| -.FSDKR(4)--KTAER(1).- | 783 | 772 | 21.38 |
| *Inter α-toroid – C-terminal domain* | | | |
| -.DMAKDYMER(4)--KTAER(1).- | 127 | 772 | 23.54 |
| -.KTAER(1)--DMAKDYMER(4).- | 772 | 127 | 23.54 |
